# Promoting tau secretion and propagation by hyperactive p300/CBP via autophagy-lysosomal pathway in tauopathy

**DOI:** 10.1186/s13024-019-0354-0

**Published:** 2020-01-06

**Authors:** Xu Chen, Yaqiao Li, Chao Wang, Yinyan Tang, Sue-Ann Mok, Richard M. Tsai, Julio C. Rojas, Anna Karydas, Bruce L. Miller, Adam L. Boxer, Jason E. Gestwicki, Michelle Arkin, Ana Maria Cuervo, Li Gan

**Affiliations:** 1 Gladstone Institute of Neurological Disease, University of California, San Francisco, CA 94158 USA; 2 Department of Neurology, University of California, San Francisco, CA 94158 USA; 3 Small Molecule Discovery Center, Department of Pharmaceutical Chemistry, University of California, San Francisco, CA 94158 USA; 4 Institute for Neurodegenerative Disease, Department of Pharmaceutical Chemistry, Weill Institute for Neurosciences, University of California, San Francisco, CA 94158 USA; 5 Memory and Aging Center, University of California, San Francisco, CA 94158 USA; 6 Department of Developmental and Molecular Biology, Albert Einstein College of Medicine, Bronx, NY 10461 USA; 7 Institute for Aging Studies, Albert Einstein College of Medicine, Bronx, NY 10461 USA; 8 Helen and Robert Appel Alzheimer’s Disease Research Institute, Brain and Mind Research Institute, Weill Cornell Medicine, New York, NY 10065 USA

**Keywords:** Tauopathy, Tau secretion, Tau spreading, Autophagy-lysosomal pathway, p300/CBP

## Abstract

**Background:**

The trans-neuronal propagation of tau has been implicated in the progression of tau-mediated neurodegeneration. There is critical knowledge gap in understanding how tau is released and transmitted, and how that is dysregulated in diseases. Previously, we reported that lysine acetyltransferase p300/CBP acetylates tau and regulates its degradation and toxicity. However, whether p300/CBP is involved in regulation of tau secretion and propagation is unknown.

**Method:**

We investigated the relationship between p300/CBP activity, the autophagy-lysosomal pathway (ALP) and tau secretion in mouse models of tauopathy and in cultured rodent and human neurons. Through a high-through-put compound screen, we identified a new p300 inhibitor that promotes autophagic flux and reduces tau secretion. Using fibril-induced tau spreading models in vitro and in vivo, we examined how p300/CBP regulates tau propagation.

**Results:**

Increased p300/CBP activity was associated with aberrant accumulation of ALP markers in a tau transgenic mouse model. p300/CBP hyperactivation blocked autophagic flux and increased tau secretion in neurons. Conversely, inhibiting p300/CBP promoted autophagic flux, reduced tau secretion, and reduced tau propagation in fibril-induced tau spreading models in vitro and in vivo.

**Conclusions:**

We report that p300/CBP, a lysine acetyltransferase aberrantly activated in tauopathies, causes impairment in ALP, leading to excess tau secretion. This effect, together with increased intracellular tau accumulation, contributes to enhanced spreading of tau. Our findings suggest that inhibition of p300/CBP as a novel approach to correct ALP dysfunction and block disease progression in tauopathy.

## Background

Tauopathies, including Alzheimer’s disease (AD) and frontotemporal lobe degeneration with tau inclusions, are neurodegenerative diseases characterized by deposition of neurofibrillary tangles composed of the microtubule-associated protein tau in the brain. Tau pathology spreads in a stereotypic pattern, which is used to stage the disease [1, 2]. Cell-to-cell transmission of pathogenic tau species may account for the spreading of tau pathology in the brain [3–5], however, the process by which tau transmits between neurons is poorly understood.

Homeostatic levels of tau are maintained by the autophagy-lysosomal pathway (ALP) and the ubiquitin-proteasomal system (UPS). Impairment of these degradative systems leads to intracellular tau accumulation, neuronal deficits, and aggregate formation [6]. Tau is also present in the cerebrospinal fluid (CSF) and interstitial fluid, and in the conditioned medium of neuronal culture. Extracellular tau species can be toxic to neurons [7, 8] and more importantly, may serve as pathogenic “seeds” to be taken up by surrounding neurons, enabling prion-like propagation [9, 10]. Rather than being passively released after neuronal death, tau is actively secreted by live neurons [11, 12]. Tau lacks a signal peptide, and may be exported by an unconventional secretion pathway [11, 13], that overlaps with ALP [14, 15]. Thus, autophagy—a key mechanism for tau degradation—could also play a role in tau secretion and propagation.

p300, a lysine acetyltransferase for numerous nuclear and cytosolic proteins, is a master regulator of Alzheimer’s disease progression [16]. Previously, we showed that p300 and its close homolog CBP directly acetylate tau, and that hyperacetylation of tau caused by increased p300/CBP activity promotes tau accumulation [17, 18]. Inhibition of p300/CBP reduces tau accumulation, tau pathology, and cognitive deficits in tau transgenic mice, highlighting a critical role for p300/CBP in tauopathies [17]. However, whether p300/CBP also contributes to tau secretion and propagation is unknown.

In this study, we show that p300/CBP activity is elevated in the setting of tauopathy, and that this is associated with dysfunction of ALP. Using HEK293T cell reconstitution and neuronal culture, we investigated the relationship between p300/CBP, autophagic flux and tau secretion. We performed a high throughput screen for new p300 inhibitors, and identified a compound that has strong effect on reducing tau secretion. Using this compound and genetic manipulations, we further show that p300/CBP inhibition affects tau propagation in both in vitro and in vivo tau spreading models.

## Materials and methods

### Primary antibodies and chemicals

Antibodies for p300 (N-15, SCBT), CBP (Cell Signaling), acetylated H3/K18 (Abcam), H3 (Cell Signaling), LC3B (Acris), LC3A (Novus Biologicals), p62/SQSTM1 (Novus Biologicals), GAPDH (Sigma), actin (DSHB), Tau-5 (Bio-Source), HT7 (Thermo Fisher), BT2 (Thermo Fisher), AT8 (Thermo Fisher), caspase-3 (Cell Signaling), α-tubulin (Abcam), MAP-2 (Millipore), GST-D2 (Cisbio), and europium cryptate-labeled anti-rabbit IgG-EuK (Cisbio) were purchased. Ac-tau/K174 antibody (AC312) and ac-tau/K274 antibody (mAB359) were produced and characterized as described previously [17,69]. The MC1 antibody was a gift from Dr. Peter Davies (Feinstein Institute). CTB (Sigma), NH_4_Cl (Sigma), Leupeptin (Sigma), vinblastine (Sigma), rapamycin (Invivogen), and Bafilomycin A1 (Invivogen) were purchased.

### Human samples

CSF samples were obtained from 13 patients with Alzheimer’s disease (AD) and 14 cognitively normal participants (Additional file 1: Table S1). Patients were recruited through the research programs: Frontotemporal Dementia: Genes, Images and Emotions, 4-Repeat Tauopathy Neuroimaging Initiative (4RTNI) and Alzheimer’s Disease Research Center (ADRC) at the University of California, San Francisco (UCSF). All patients received a comprehensive neurological history, physical, structured caregiver interview and neuropsychology assessment. Diagnosis was made by consensus panel, utilizing the standard diagnostic criteria for AD and PSP [19, 20]. Cognitively normal healthy patients were recruited through the Larry L. Hillblom Aging Study at UCSF. The eligibility criteria included ages 60–100, with no significant subjective memory complaints, no functional impairment, a Clinical Dementia Rating (CDR) of 0, no diagnosis of mild cognitive impairment, and a Mini-Mental State Exam (MMSE) score of ≥26.

CSF collection and processing was performed according to the Alzheimer’s Disease Neuroimaging Initiative protocol. CSF was obtained by lumbar puncture using a 25-gauge needle and collected in 10-mL polypropylene tubes. Within 1 h, CSF was centrifuged at 2000 g for 10 min at 4 °C, transferred to new polypropylene tubes and stored at − 80 °C until analysis.

### Protein purification

DNA encoding a fragment of p300 (aa 1815–1910), including the PHD, catalytic (HAT), ZZ and TAZ2 domains, was cloned into a pET24b vector. The plasmid was transformed into the *E. coli* Rosetta BL21 strain (Invitrogen). Frozen cell stock was streaked onto a Kanamycin (50 μg/mL) plate and grown overnight. One colony was picked and grown in a starter culture and used to inoculate 6 L of 2X YT media. Upon log-phase growth (OD ~ 0.6–0.8), expression was carried out by overnight induction with 0.2 mM IPTG at 16 °C. The cells were harvested at 5000 rpm for 15 min and resuspended in 100 mM NaCl, 100 mM Tris pH 8.0 and disrupted through a microfluidizer. The lysate was then spun down at 20,000 rpm for 45 min and filtered. Protein was purified in two steps by Ni affinity chromatography and anion exchange chromatography using an ÄKTA system (GE Healthcare). The lysate was then loaded onto a 1 mL HisTrap HP column (GE Healthcare). The column was subsequently washed with 10% B and 20% B and eluted with 100% B. The Ni elution fraction was diluted 10-fold with 20 mM Tris pH 8.0 and was loaded onto a 1 mL HiTrap Q column (GE Healthcare). Elution was carried out by a 0–100% B gradient over 20 column volumes collecting 1.0 mL fractions. Flow rates were typically held constant at 1.0 mL/min or lowered if the pressure exceeded the limit of the column accordingly. HitrapQ fractions were further polished on gel filtration column superdex 200 16/60 in 20 mM Tris pH 8.0, 150 mM NaCl. GST-tau was produced as previously reported [18].

### HTS of p300 inhibitors based on the homogeneous time-resolved fluorescence assay

50 nL of compound (final 0.5% DMSO) was added to 5 μL (final 6 nM) GST-tau in a 384-well plate. The reaction was initiated by adding 5 μL (final 1 nM) p300, followed by 1 h incubation at RT. At the end of the reaction, 10 μL/well of quench/detection mixture containing 10 nM mAB359, 2.4 nM donor (anti-rabbit IgG-EuK), 3.6 nM acceptor (anti-GST-D2), and 25 μM anacardic acid (a known p300 inhibitor as the quench reagent) in detection buffer (50 mM sodium phosphate, pH 7.9, 0.8 M KF) was added. The final mixture was then incubated at RT for another 2 h. After incubation, signal was read on EnVision Multilabel Plate Reader (PerkinElmer; ex: 340 nm, em: 665/620 nm). DMSO and anacardic acid served as negative and positive controls, respectively. Percent inhibition was calculated as (FRET signal of DMSO-FRET signal of compound)/(FRET signal of DMSO-FRET signal of anacardic acid) × 100%.

To rule out compounds that suppress FRET through mechanisms other than inhibition of p300, a counter screen was performed in which compounds were added after 1 h incubation of the enzymatic reaction, followed immediately by the subsequent quench/detection. Any compounds that suppressed the FRET signal were considered false positives.

### Orthogonal MMBC assay

The thiol-reactive dye MMBC ([10-(2,5-dihydro-2,5-dioxo-1H-pyrrol-1-yl)-9-methoxy-3-oxo-,methyl ester 3H-naphthol (2,1-b) pyran-S-carboxylic acid, known as ThioGlo 1] was used to detect CoA, the product of the acetylation reaction. Once MMBC reacts with CoA, it becomes fluorescent with excitation wavelength at 379 nm and emission wavelength at 513 nm. The assay was performed with a final volume of 50 μL in assay buffer (100 mM HEPES, pH 7.5, 0.01% Triton-X100, 500 μM TCEP). Compounds of various concentrations were added to the mixture of 200 nM p300, 0.6 μM Tau, 100 μM Ac-CoA, and 250 μM MMBC. The reaction progress was continuously monitored on plate reader Flexstation III (Molecular Devices) for 1 h. The slopes of the linear portion of the reaction were used to calculate the percentage of inhibition.

### Differential scanning Fluorimetry

1 μM p300 with 5X syprorange was incubated with 25 μM, or 50 μM 37892 in the assay buffer. The T_m_ of p300 was monitored on real-time PCR machine Stratagene Mx3005p.

### HEK293T cell culture

HEK293T cells cultured in Dulbecco’s Modified Eagle’s medium (DMEM) with 10% fetal bovine serum were transfected with expression vectors encoding FLAG-tagged full-length hTau and myc-tagged p300, or empty vector, using lipofectamine 2000 (Life Technology). For mCherry-GFP-LC3 reporter, HEK293T cells were infected with lentivirus expressing mCherry-GFP-LC3, and a single clone of infected cells with moderate expression was isolated to establish the reporter line.

### Primary neuronal cultures and Lentiviral or AAV infections

Primary neuronal cultures were established from cortices of Sprague-Dawley rat (Charles River Laboratories) or mouse pups on postnatal day 0 or 1. Purified cells were plated at 600,000 cells/ml in Neurobasal medium supplemented with B27 (Invitrogen) on poly-ornithine-coated plates. All experiments were performed at 11–14 days in vitro (DIV) unless noted otherwise. Lentivirus was generated, purified, and used for infection as described [22]. Briefly, recombinant lentivirus was produced by cotransfection of the shuttle vector (pRRL), two helper plasmids, delta8.9 packaging vector, and VSV-G envelope vector into HEK293T cells and purified by ultracentrifugation [23]. AAV viral vectors expressing P301S tau were generated and purified from Viro-vek, Inc. (Hayward, CA). All drug treatments and analyses were performed at DIV 10–13 days.

### Differentiation of human iPSC–derived neurons

Human iPSC-derived neurons were differentiated with a simplified two-step protocol (pre-differentiation and maturation), as described previously [41]. Briefly, iPSCs (WTC11 line) containing a *Ngn2* transgene integrated into the AAVS1 locus were pre-differentiated in Knockout Dulbecco’s modified Eagle’s medium (KO-DMEM)/F12 medium containing growth factors and doxycycline. After pre-differentiation, the neuronal precursor cells were matured in maturation medium containing 50% DMEM/F12, 50% Neurobasal-A medium with all the growth factors. Human neurons were assayed at 5–8 weeks post-differentiation.

### Tau secretion assay

Neuronal tau secretion assay was performed on mature neurons (DIV 11–14 for primary neurons, 5–8 weeks old for human iPSC-derived neurons). Fresh artificial cerebrospinal fluid (ACSF) containing NaCl (140 mM), KCl (5 mM), CaCl_2_ (2.5 mM), MgCl_2_ (2 mM), HEPES (10 mM) and glucose (10 mM) was added to neurons to replace the original medium (neurobasal medium with B27 supplement). After 3 h incubation at 37 °C, the conditioned medium was harvested and centrifuged at 8000 RPM to remove cell debris, and subjected to tau ELISA analysis to quantify the amount of extracellular tau. To analyze the truncation of extracellular tau, the conditioned medium was concentrated by 12.5-fold using Amicon Ultra-0.5 Centrifugal Filter Unit with Ultracel-3K membrane (Millipore), and subjected to immunoblot analysis. For HEK293T cells, serum-free DMEM was used to replace post-transfection medium, and incubated at 37 °C for 24–48 h. The conditioned medium was harvested and centrifuged at 8000 RPM to remove cell debris, and concentrated by 25-fold using Amicon Ultra-0.5 Centrifugal Filter Unit with Ultracel-30K membrane (Millipore). The concentrated medium was subjected to immunoblot analysis to quantify the amount of extracellular t-tau, p-tau and ac-tau. Tau secretion was calculated by normalizing levels of extracellular tau (in the conditioned medium) to the intracellular tau (in the lysate). LDH assay (Promega) was used to monitor cell toxicity per manufacturer’s protocol.

### Homogenization of cells and tissues for Immunoblot analyses

HEK293T cells or mouse brain tissues were homogenized in RIPA buffer containing protease inhibitor cocktail (Sigma), 1 mM phenylmethyl sulfonyl fluoride, phosphatase inhibitor cocktail (Sigma), 5 mM nicotinamide (Sigma) and 1 μM trichostatic-A (Sigma). Neurons were homogenized in N-Per buffer (Thermo Fisher) containing all the inhibitors as above. Mouse brain tissues were sonicated after homogenization. Lysates were centrifuged at 14,000 RPM at 4 °C for 15 min. Supernatants were collected and protein concentrations were determined by the BCA assay (Thermo Fisher). Pellets containing the nuclei were washed with 1/3 volume of the lysis buffer, and used for histone extraction by overnight incubation with HCl (0.24 N). The same amount of protein was resolved on a 4–12% SDS-PAGE gel (Invitrogen), transferred to PVDF membrane (Bio-Rad), and probed with appropriate antibodies. Bands in immunoblots were visualized by enhanced chemiluminescence (Pierce) and quantified by densitometry and ImageJ software (NIH). Representative blots from same gel/membrane are shown and compared in the same figure. Samples from non-adjacent lanes are separated by a line.

### ELISA

AcH3K18 ELISA was performed using EpiQuik Global Acetyl Histone H3K18 Quantification Kit (Colorimetric) (EpiGentek). CSF samples were concentrated by 10-fold using Amicon Ultra Centrifugal Filter Unit with Ultracel-3K membrane (Millipore). Mouse β-tubulin ELISA was performed using Mouse TUB β ELISA kit (MyBiosource MBS723313), according to manufacturer’s protocol. Mouse Aβ40 and Aβ42 ELISA was performed using Amyloid β 40 and Amyloid β 42 Mouse ELISA Kit (Thermo Fisher), according to manufacturer’s protocol. Sensitive Tau ELISAs were adapted and modified according to previous reports [24, 25] and as described previously [41]. Briefly, mouse monoclonal antibody BT2 or HT7 was used for capture. The respective analytes were detected with alkaline phosphatase–conjugated mouse monoclonal antibody against Tau5 (BioLegend). Recombinant full length hTau (rPeptide) was used to generated standard curves for each assay. The CDP-Star substrate (Invitrogen) was used as chemiluminescent alkaline phosphatase substrate.

### Immunofluorescence staining

Cells grown on coverslips were washed with phosphate-buffered saline (PBS) and fixed in fresh 4% paraformaldehyde, and permeabilized with 0.1% Triton X-100. Coverslips were washed in PBST (phosphate-buffered saline, 0.01% Triton X-100), and incubated for 1 h in blocking solution containing PBST and 5% normal goat serum. The cells were then incubated in blocking solution containing primary antibody overnight at 4 °C, followed by incubation with secondary antibody for 1 h. Primary antibodies include MC1 (1:500), MAP2 (1:800) and acH3K18 (1:1000). Secondary antibodies include fluorescein-labeled goat anti-mouse IgG and goat anti-rabbit IgG (1:500, Vector Laboratories). Hoechst (Thermo Fisher) was used to label the nuclei. Images were acquired by Leica microscope (DM5000 B) and analyzed by Micro-Manager software (UCSF, San Francisco, CA). For *p300*^*F/F*^*/CBP*^*F/F*^ and *p300*^*F/+*^*/CBP*^*F/+*^ neurons, a fully automated ArrayScan high-content system (Thermo) was used to acquire images and quantify MC1 signal and neuronal health parameters.

### RNA isolation and quantitative real-time PCR (qRT-PCR)

Total RNA was isolated from *p300*^*F/F*^*/CBP*^*F/F*^ primary neurons with the Direct-zol™ RNA MiniPrep kit (Zymo), and the remaining DNA was removed by incubation with RNase-free DNase (Zymo). Purified messenger RNA was then converted to complementary DNA by the TaqMan reverse transcription (RT) kit (Applied Biosystems). Quantitative RT-PCR was performed on the ABI 7900 HT sequence detector (Applied Biosystems) with SYBR Green PCR master mix (Applied Biosystems). The average value of three replicates of each sample was expressed as the threshold cycle (Ct), at which the fluorescence signal starts to increase rapidly. Then, the difference between the Ct value for hTau and the Ct value for mouse GAPDH (ΔCt = Ct (hTau)-Ct (GAPDH)) was calculated for each sample. The relative levels of gene expression for each sample was determined by 2^-ΔCt^ and expressed as the fold change. The following primers were used for quantitative RT-PCR: hTau (forward, 5′-GTTGGGGGACAGGAAAGATCAG-3′; reverse, 5′-CCGGGAGCTCCCTCATC-3′), mouse GAPDH (forward, 5′-GGGAAGCCCATCACCATCTT-3′; reverse, 5′-GCCTTCTCCATGGTGGTGAA-3′).

### Tau purification and fibrilization

Myc-tagged repeat domain (K18) of P301L tau was expressed in Terrific Broth media containing sodium chloride (500 mM) and a small-molecule chaperone, betaine (10 mM), to improve expression and minimize degradation. Expression was induced with 200 μM IPTG for 3.5 h at 30 °C. tau was purified as described [26]. Purified tau was dialyzed overnight at 4 °C into aggregation assay buffer (Dulbecco’s PBS pH 7.4, 2 mM MgCl_2_, 1 mM DTT). Aggregation of tau (20 μM) was induced by the addition of a freshly prepared heparin sodium salt solution (Santa Cruz) at a final concentration of 88 μg/ml. Tau fibril samples were labeled with Alexa Fluor 647 NHS ester (Molecular Probes) at 1:40 M ratio of dye:tau monomer for 1 h at room temperature. Samples were centrifuged at 100,000 g for 1 h at 4 °C to remove unreacted free dye. Pellets containing tau fibrils were resuspended in Dulbecco’s PBS, pH 7.4, 2 mM MgCl_2_. Tau fibril concentrations were quantified by SDS-PAGE with a set of tau monomer standards.

### In vitro fibril-induced tau spreading model

The in vitro fibril-induced tau spreading model was adapted and modified from previously described [27]. Briefly, primary neurons cultured on coverslips were infected with AAV-P301S hTau on DIV3, and synthetic tau fibrils (K18/P301L, 100 nM) were added to the medium on DIV5. Neurons without AAV infection (AAV-) and neurons treated with PBS instead of fibrils were included as negative controls. Neurons were incubated with the fibrils for 7–10 days. To test the effect of compound treatment, drugs were added to the culture medium together with the fibrils, and replenished once after 3 days. Immunofluorescence staining using the MC1 antibody (1:500) was performed to detect tau aggregates. Briefly, neurons grown on coverslips were washed with fresh medium containing 0.01% trypsin, and fixed in fresh 4% PFA with 0.1% Triton-X, followed by regular immunofluorescence staining procedure.

### Immunohistochemistry and image analysis

Anesthetized mice were transcardially perfused with 0.9% saline. Mouse brains were removed and fixed in 4% paraformaldehyde for 48 h. Fixed brains were cryopreserved in 30% sucrose in PBS for at least 2 days, and coronal brain sections (30 μm) were obtained with a sliding microtome. For immunostaining, floating brain sections were permeabilized and incubated in blocking solution (10% normal goat serum in 0.3% Triton X-100 TBST) at room temperature for 1 h. Sections were then incubated with primary antibodies, including MC1 (1:500), acH3K18 (1: 1000) and Dapi. After overnight incubation, the sections were incubated with secondary antibodies including Cy3-labeled donkey anti-rabbit IgG (1:500, Jackson ImmunoResearch) and fluorescein-labeled goat anti-mouse IgG (1:500, Vector Laboratories). Images were acquired by Keyence BZ-X7000 microscope, and analyzed using ImageJ software (NIH). Experimenters quantifying immunoreactivity were blind to the mouse genotype and treatment conditions.

### Mice

Mice were housed in a pathogen-free barrier facility with a 12-h light/dark cycle and ad libitum access to food and water. All animal procedures were carried out under guidelines approved by the Institutional Animal Care and Use Committee of the University of California, San Francisco. P301ShTau transgenic mice (PS19), *Ep300*^*F/F*^ and *CBP*^*F/F*^ mice were purchased from Jackson Laboratory. *Ep300*^*F/F*^ mice were crossed with *CBP*^*F/F*^ mice to generate *Ep300*^*F/F*^*/CBP*^*F/F*^ mice. PS19 mice were crossed to *Ep300*^*F/F*^*/CBP*^*F/F*^ mice to generate *P301ShTau/p300*^*F/+*^*/CBP*^*F/+*^ mice, which were further crossed with *Ep300*^*F/F*^*/CBP*^*F/F*^ mice to generate *P301ShTau/Ep300*^*F/F*^*/CBP*^*F/F*^ mice. Mice were assigned into gender- and age-matched treatment groups in a randomized manner for all experiments. The sample size for each experiment was determined based on previous experiences with these models.

### Stereotaxic injection

Mice were anesthetized with 2% isoflurane by inhalation for the duration of surgery, and secured on a stereotaxic frame (Kopf Instruments). 3–4-month-old PS19 mice and non-transgenic control mice were injected stereotaxically at a rate of 0.5 μl/min, with equal amounts (1 × 10^12^ genomic particles) of AAV1 expressing either Cre or GFP control (ViroTek), into the dentate gyrus of right hippocampus. 2 μL of 3.5 mg/ml synthetic tau fibrils (K18/PL) were injected into CA1 region of left hippocampus. The following coordinates were used for dentate gyrus (anterior-posterior − 2.1, medial-lateral − 1.7, dorsal-ventral − 2.1) and CA1 (anterior-posterior − 2.5, medial-lateral + 2.0, dorsal-ventral − 1.8). Control animals were injected with 2 μL of PBS instead of fibrils.

### Statistics

Data were analyzed with GraphPad Prism 7 (GraphPad) or Stata. Differences between means were assessed with paired or unpaired Student’s *t* test, one-way or two-way analysis of variance, followed by post hoc testing of pairwise comparisons among genotypes (with Tukey’s or Dunnett’s correction for one-way ANOVA and Bonferroni correction for two-way ANOVA), as indicated. Pearson’s correlation coefficients were used to quantify the linear relationship between two variables. Outliers are pre-established as data outside of mean ± 2SD. The level of significance was set as *p* < 0.05.

## Results

### Hyperactive p300/CBP in tauopathy brains is associated with ALP impairment

**Fig. 1:**
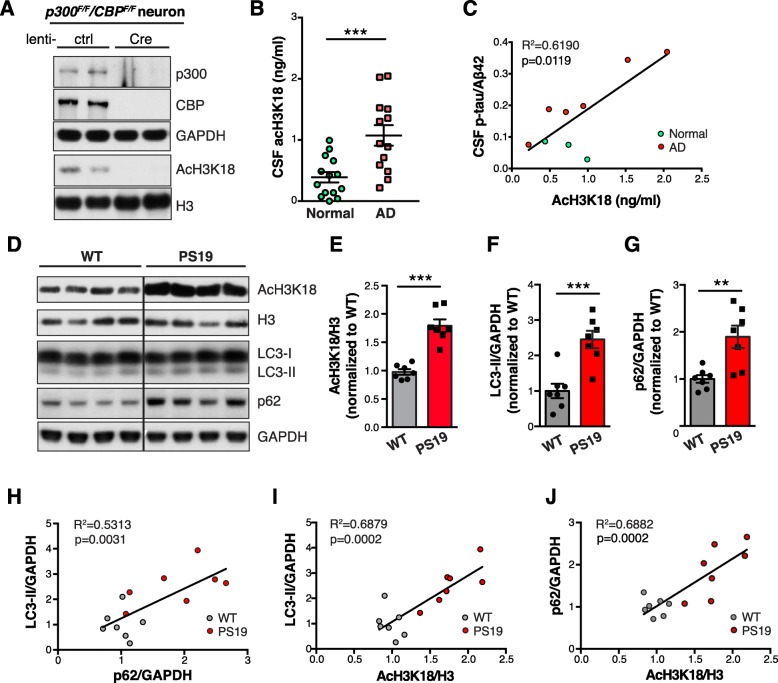
Hyperactive p300/CBP Is Associated with Impairment in the Autophagy-Lysosomal Pathway in Tauopathy Brains **a** Representative immunoblots of p300, CBP, acH3K18, and H3 in lysates of *p300*^*F/F*^*/CBP*^*F/F*^ primary neurons infected with lenti-control (ctrl) or lenti-Cre. **b** Levels of acH3K18, measured by ELISA, in CSF samples from normal controls (normal, *n* = 14) and patients with AD (*n* = 13). ***p < 0.001 by unpaired *t* test. Values are mean ± SEM. **c** Pearson correlation analysis of acH3K18 and CSF p-tau/Aβ42 levels in CSF samples from normal controls (normal, *n* = 3) and AD patients (*n* = 6). **d** Representative immunoblots of acH3K18, total H3, LC3-I, LC3-II, SQSMT/p62, and GAPDH in hippocampal lysates of 10-month-old wildtype (WT) and PS19 mice. **e**–**g** Levels of acH3K18 relative to total H3 (**e**), LC3-II (**f**) and p62 (**g**) relative to GAPDH, normalized to WT. **h**–**j** Pearson correlation analysis of LC3-II and p62 levels (**h**), acH3K18 and LC3-II levels (**i**), and acH3K18 and p62 levels (**j**). (**d**–**j**) *n* = 7 mice per group. **p < 0.01, ***p < 0.001 by unpaired *t* test. Values are mean ± SEM

To investigate the role of p300/CBP in the pathogenesis of tauopathies, we first evaluated the activity of p300/CBP in healthy and diseased brains. Acetylation of the p300/CBP target –histone H3– at lysine 18 (acH3K18) [28] was completely absent in p300/CBP double-knockout primary neurons (Fig. 1a and Additional file 1: Figure S1*A*), making it a specific readout for neuronal p300/CBP activity. ELISA demonstrated significantly higher acH3K18 in CSF samples from patients with AD than in healthy controls (Fig. 1b). This increase is significantly correlated with CSF levels of p-tau/Aβ42, a diagnostic biomarker of AD [29] (Fig. 1c).

**Fig. 2:**
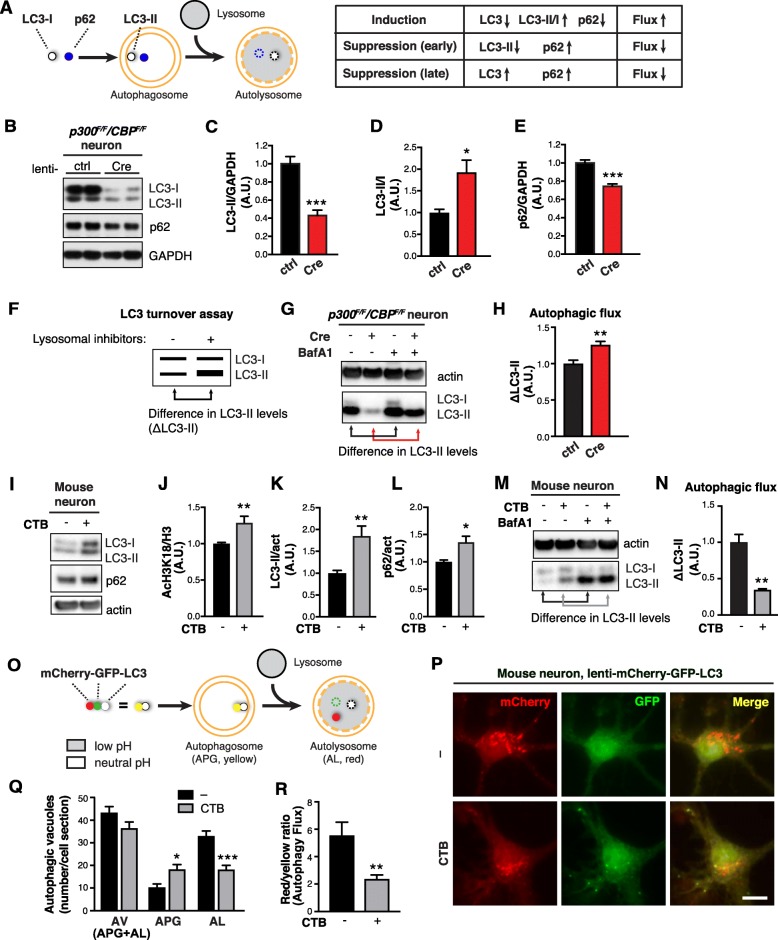
p300/CBP Inhibits Autophagic Flux in Neurons **a** Schematic diagram showing autophagic flux with changes in autophagic markers. **b**–**e** p300/CBP double knockout in primary mouse neurons reduces LC3 and p62 accumulation. **b** Representative immunoblots of LC3-I, −II, p62, and GAPDH in lysates of *p300*^*F/F*^*/CBP*^*F/F*^ primary neurons infected lenti-ctrl or lenti-Cre. **c**–**e** Quantification of LC3-II (**c**), p62 (**e**) relative to GAPDH, and LC3-II/I (**d**), normalized to control. *n* = 6 wells from three independent experiments. *p < 0.05, ***p < 0.001 by unpaired *t* test. **f** Measuring autophagic flux by LC3 turnover assay. **g**, **h** p300/CBP double knockout in primary mouse neurons increases autophagic flux. **g** Representative immunoblots of LC3-I, −II and actin in lysates of p300/CBP double knockout neurons that were treated with BafA1 (10 nM) or DMSO for 24 h. **h** Quantification of autophagic flux based on the difference (increase) of LC3-II in response to BafA1 treatment, normalized to control (Cre-, BafA1-). *n* = 4 wells from two independent experiments. **p < 0.01 by unpaired *t* test. **i**–**n** CTB treatment in primary neurons reduces autophagic flux. **i** Representative immunoblot of LC3-I, −II, p62 and actin in lysates of primary mouse neurons treated with DMSO (control) or CTB (50 uM) for 24 h. **j**–**l** Quantification of acH3K18 relative to H3 (**j**), LC3-II (**k**) and p62 (**l**) relative to actin, normalized to control. *n* = 6 wells from four independent experiments. *p < 0.05, **p < 0.01 by unpaired *t* test. **m** Representative immunoblot of LC3-I, −II and actin in lysates of primary mouse neurons treated with DMSO (control), CTB (50 uM), BafA1 (10 nM), or CTB + BafA1 for 24 h. **n** Quantification of autophagic flux by the difference (increase) of LC3-II in response to BafA1, normalized to control (CTB-, BafA1-). *n* = 3 wells from three independent experiments. **p < 0.01 by unpaired *t* test. **o** Using mCherry-GFP-LC3 color change to measure autophagic flux. **p** Representative images of primary mouse neurons infected with lentivirus expressing mCherry-GFP-LC3 treated with CTB (50 uM) or DMSO (control) for 24 h. Scale bar: 10 μm. **q** Quantification of autophagic vesicles (AV). Autophagosomes (APG) are identified as yellow vesicles retaining both mCherry and GFP fluorescence. Autolysosomes (AL) are identified as red vesicles in which GFP fluorescence is quenched by the low pH in lysosomes. *p < 0.05, ***p < 0.001, two-way ANOVA, Tukey-Kramer post hoc analysis. **r** Ratio of the number of red vesicles to yellow vesicles per cell. **p < 0.01, unpaired *t* test. From two independent experiments, *n* = 12 cells (control), *n* = 17 cells (CTB). Values are mean ± SEM

AD brains with high tau burden exhibit impaired ALP and accumulate undegradable autophagic vacuoles (AVs), which are rare in healthy brains [30–32]. Because p300 has been reported to regulate autophagy [33, 34], we investigated the relationship between p300/CBP and ALP impairment in tauopathy. In line with our observations in human patients, acH3K18 levels were significantly higher in the hippocampi of 10-month-old tauP301S transgenic mice (PS19), a model of tauopathy [35], than in wild-type (WT) littermates (Fig. 1d and e), indicating p300/CBP hyperactivity. Such difference was also observed in 3-month-old PS19 mice and WT littermates (Additional file 1: Figure S1 *C* and *D*), and p300/CBP hyperactivity in PS19 mice maintained at the same level as the mice age (Additional file 1: Figure S1 *E* and *F*). Levels of autophagosome markers LC3-II and SQSTM1/p62 were increased in PS19 mice compared to WT littermates (Fig. 1 d, f and g). Since LC3-II and p62 are degraded in autolysosomes upon autophagosome maturation, higher steady-state levels of these markers suggest an accumulation of immature AVs (Fig. 2a), consistent with previous immuno-electron microscopy studies [30, 31]. Interestingly, levels of LC3-II and p62 correlate with each other (Fig. 1h), and each correlated with p300/CBP activity (Fig. 1 i and j), implicating an underlying relationship between hyperactive p300/CBP and ALP impairment in tauopathy brains.

### p300/CBP inhibits autophagic flux

To directly examine how p300/CBP affects ALP in neurons, we measured autophagic markers in p300/CBP double knockout neurons. In neurons with postmitotic knockout of p300 and CBP, we observed significantly reduced steady-state levels of LC3-I, LC3-II, and p62 (Fig. 2 b, c, e), suggesting enhanced autophagic flux (Fig. 2a). We measured autophagic flux by LC3 turnover assay (Fig. 2f). Upon bafilomycin A1 (BafA1) treatment, p300/CBP knockout neurons accumulated more LC3-II from baseline compared to WT neurons (Fig. 2g, h). Thus, the reduction of steady-state LC3-II in p300/CBP knockout neurons was due to enhanced autophagic flux, rather than to reduced induction of autophagy. In agreement with this, the conversion of LC3-I to LC3-II was accelerated in p300/CBP knockout neurons as reflected by the LC3-II/I ratio (Fig. 2d). We next examined autophagic flux in response to p300/CBP activation in primary neuronal culture. Treatment with a small molecule p300/CBP activator CTB [36] increased p300/CBP activity as expected (Fig. 2j), increased steady-state levels of LC3-II and p62 (Fig. 2 i, k and l), and reduced autophagic flux as measured by LC3 turnover assay with BafA1 co-treatment (Fig. 2 m and n). In addition, we used a dual-labeled fluorescent reporter, mCherry-GFP-LC3, to monitor the maturation of AVs [37]. In this system, immature AVs with neutral pH are yellow since both mCherry and GFP are fluorescent, whereas mature AVs with acidic pH are red since only mCherry retains fluorescence (Fig. 2o). In control neurons expressing mCherry-GFP-LC3, there were more red AVs than yellow AVs (Fig. 2 p and q), indicating that most AVs were mature and autophagic flux was normal. In contrast, CTB treatment significantly increased the number of yellow AVs and reduced the number of red AVs (Fig. 2 p and q), resulting in a reduced red-to-yellow ratio (Fig. 2r). Similar results were observed in HEK293T cells overexpressing p300 and tau: Overexpression of p300 increased steady-state levels of both LC3-II and p62 (Additional file 1: Figure S2, *A* and *B*), and reduced autophagic flux measured by LC3 turnover assay (Additional file 1: Figure S2, *C* and *D*). In HEK293T cells with mCherry-GFP-LC3 reporter, p300 overexpression led to reduced red-to-yellow ratio (Additional file 1: Figure S2 *E*–*G*). The reduction in the number of AVs in HEK293T cells with p300 overexpression might reflect impaired AV formation, in line with a previous report [33]. The slightly different phenotype between primary neurons and HEK293T cells is likely due to differences in cell-type-specific basal levels of autophagy. Taken together, these data indicate that p300/CBP hyperactivity inhibits autophagic flux.

### p300/CBP promotes tau secretion

**Fig. 3:**
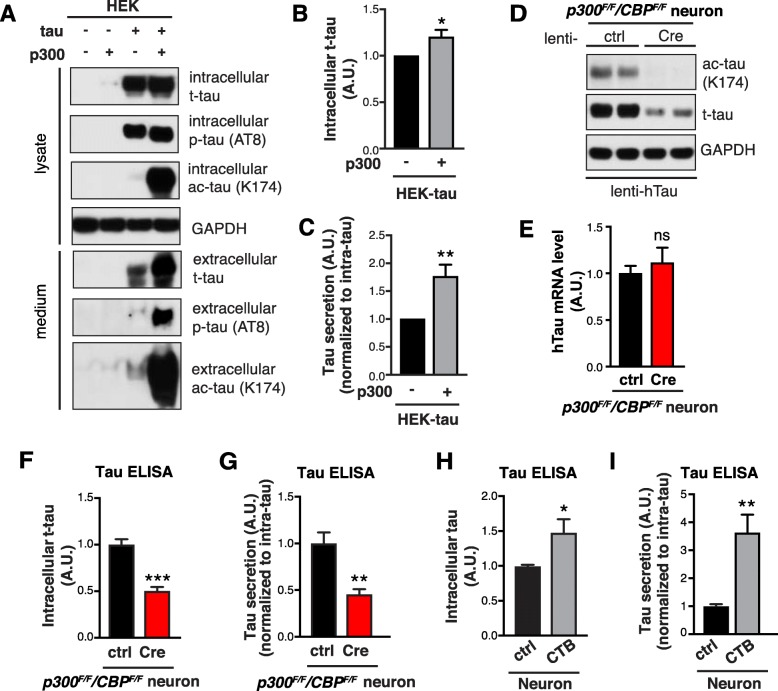
p300/CBP Promotes Tau Secretion **a**–**c** p300 overexpression in HEK293T cells increases tau secretion. **a** Representative immunoblot of total tau (t-tau), phosphor-tau (p-tau, AT8), acetylated tau (ac-tau, K174), and GAPDH in the lysate (5% of total) and conditioned medium (25% of total) of HEK293T cells transfected with tau alone or tau+p300. HEK293T cells were serum-starved for 48 h after transfection. The conditioned medium was concentrated 25-fold. **b**, **c** Quantification of intracellular total tau (t-tau) levels (**b**) and t-tau secretion (**c**), normalized to control. t-tau secretion was quantified by normalizing extracellular tau to intracellular tau. *n* = 8 wells from eight independent experiments. *p < 0.05, **p < 0.01 by unpaired *t* test. **d**–**g** p300/CBP double knockout in primary mouse neurons reduces tau secretion. **d** Representative immunoblot of ac-tau (K174), t-tau, and GAPDH in the lysate of *p300*^*F/F*^*/CBP*^*F/F*^ primary neurons infected with lenti-ctrl or lenti-Cre. **e** hTau mRNA levels in *p300*^*F/F*^*/CBP*^*F/F*^ primary neurons infected with lenti-ctrl or lenti-Cre, by qRT-PCR. *n* = 4 wells from two independent experiments. ns, non-significant by unpaired *t* test. **f** Quantification of intracellular t-tau levels by tau ELISA, normalized to control. **g** Quantification of tau secretion over 3 h by ELISA, normalized to intracellular tau levels and normalized to control. *n* = 6 wells from three independent experiments. **p < 0.01, ***p < 0.001, unpaired *t* test. **h, i** CTB treatment increase tau secretion in primary mouse neurons. **h** Quantification of intracellular total tau levels by tau ELISA, normalized to control. **i** Quantification of tau secretion over 3 h by ELISA, normalized to intracellular tau levels and normalized to control. *n* = 8 wells from four independent experiments. *p < 0.05, **p < 0.01, unpaired *t* test. Values are mean ± SEM

Tau is thought to be released through an unconventional secretion pathway [11, 12], which overlaps with ALP [14, 15, 38]. Because hyperactive p300/CBP impairs ALP and reduces autophagic flux, we investigated whether p300 hyperactivation affects tau secretion. In HEK293T cells overexpressing human tau (hTau), extracellular tau was readily detectable in the conditioned medium and was largely unphosphorylated (Fig. 3a*,* 3^rd^ lane), consistent with previous reports [39, 40]. Co-expression of p300 markedly increased the level of acetylated tau (ac-tau) and increased intracellular accumulation of total tau (t-tau) (Fig. 3 a and b), as expected, since hyperacetylation of tau is known to increase its stability and promote accumulation [17, 18]. Remarkably, p300 overexpression also led to significantly increased t-tau secretion (measured as levels of extracellular tau normalized to intracellular tau) (Fig. 3a and c), and increased the levels of secreted phosphorylated tau (p-tau, AT8-positive) (Fig. 3a*,* 4^th^ lane). We then examined how genetic inhibition of p300/CBP affects tau in neurons. In p300/CBP double-knockout neurons overexpressing hTau, tau acetylation was abolished as expected (Fig. 3d), and intracellular t-tau levels were reduced without affecting tau mRNA levels (Fig. 3e and f), consistent with decreased protein stability associated with reduced acetylation [17]. Strikingly, p300/CBP knockout drastically reduced tau secretion (Fig. 3g), supporting the conclusion that p300/CBP promotes tau secretion. Indeed, when neurons were treated with the p300/CBP activator CTB, both intracellular tau accumulation and extracellular tau secretion were significantly increased (Fig. 3 h and i). Western blot analysis of the conditioned media showed that CTB treatment increased the secretion of not only full-length tau, but also truncated tau (Additional file 1: Figure S3 *A*). The increased tau release mainly resulted from physiological secretion rather than cell damage or death, as we detected minimal caspase-3 cleavage and no tubulin release associated with CTB treatment (Additional file 1: Figure S3, *B*–*D*). In addition, CTB treatment did not alter the secretion of Aβ40 and Aβ42 (Additional file 1: S3 *E* and *F*), suggesting that p300/CBP activation specifically affects tau secretion.

### A new p300 inhibitor reduces tau secretion

**Fig. 4:**
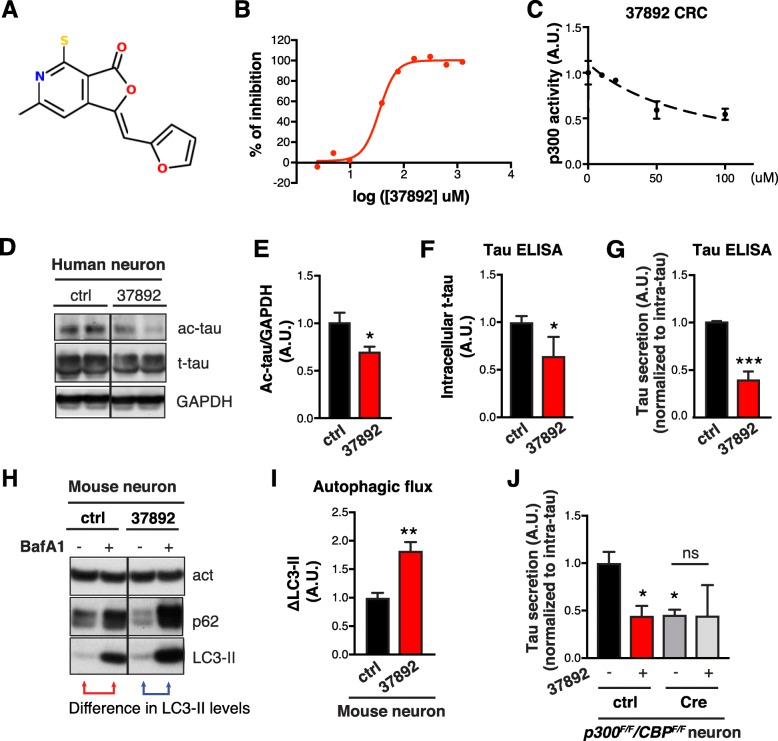
New p300 Inhibitor 37892 Reduces Tau Secretion (**a**) Structure of SMDC37892. **b** 37892 inhibited tau acetylation by p300 with an IC_50_ of 35 μM. **c** Concentration-response curve (CRC) for 37892, quantified from p300 activity in HEK293T cells. **d**–**g** 37892 treatment in human iPSC-derived neurons. **d** Representative immunoblot of ac-tau (K174), t-tau, and GAPDH in lysates of human iPSC-induced neurons treated with DMSO (control) or 37892 (50 μM) for 3 days. Quantification of levels of ac-tau (**e**) and intracellular t-tau (**f**) after 3 days of treatment, normalized to control. **g** Quantification of tau secretion over 3 h, normalized to intracellular tau levels, and normalized to control. *n* = 6 wells from three independent experiments. *p < 0.05, ***p < 0.001 by unpaired *t* test. **h**–**j** 37892 treatment increases autophagic flux in hTau-expressing mouse primary neurons. **h** Representative immunoblots of p62, LC3-II and actin in primary neurons treated with 37892 (50 μM), BafA1 (5 nM), or both, for 24 h. **i** Autophagic flux in control and 37892-treated neurons quantified from the increase of LC3-II levels in response to BafA1 treatment, normalized to control. *n* = 6 wells from three independent experiments. **p < 0.01 by unpaired *t* test. **j** Quantification of tau secretion over 3 h in p300/CBP double knockout neurons treated with 37892. *p300*^*F/F*^*/CBP*^*F/F*^ primary neurons were infected with lenti-ctrl or lenti-Cre, and treated with DMSO (−) or 37892 (50 μM) for 24 h. *n* = 4 wells from 2 independent experiments. *p < 0.05, ns, non-significant, by one-way ANOVA and Sidak’s multiple comparisons test. Values are mean ± SEM

Next, we screened 133,000 compounds from UCSF’s Small Molecule Discovery Center library for small-molecule inhibitors of p300, using a high-throughput homogeneous time-resolved fluorescence assay to measure the acetylation of full-length 2N4R tau by the purified core domain of p300 in the presence of acetyl-CoA (Additional file 1: Figure S4 *A*). Of these compounds, 1120 had inhibition values > 3 SD above the mean and were not promiscuous pan-assay interference compounds or quenchers of the donor. After validation and counter-screening to filter out fluorescence interference compounds, we confirmed 212 hits, of which 28 had an IC_50_ < 50 μM and a hillslope < 3. Twenty of the 28 compounds were commercially available and purchased for retesting (Additional file 1: Figure S4 *B*). One of the top hits, 37,892 (Fig. 4a), had an IC_50_ of 35.3 μM (Fig. 4b). Orthogonal MMBC assay confirmed its inhibitory activity (Additional file 1: Figure S4 *C*). 37892 competed with tau, as its IC_50_ values increased with increasing tau concentrations (Additional file 1: Figure S4 *D*). The binding of 37892 and p300 was determined by differential scanning fluorimetry (Additional file 1: Figure S4 *E*). In HEK239T cells, 37892 reduced p300/CBP activity (measured by acH3K18) in a dose-dependent fashion (Additional file 1: Figure S4 *F*, Fig. 4c), and did not induce significant toxicity (Additional file 1: Figure S4 *G*).

We next examined the effects of 37892 on tau homeostasis in human iPSC-derived excitatory neurons [41]. 37892 reduced intracellular ac-tau and t-tau accumulation (Fig. 4 d–f), and reduced tau secretion (Fig. 4g). On the other hand, 37892 increased autophagic flux as measured by LC3 turnover after co-treatment with BafA1 (Fig. 4 h and i). To determine whether the effect of 37892 on tau secretion is dependent on p300/CBP inhibition, we treated p300/CBP double knockout neurons with 37892, and failed to see further decrease of tau secretion in the knockout neurons (Fig. 4j). Therefore, 37892 reduces tau secretion through inhibition of p300/CBP.

### Blocking autophagic flux increases tau secretion and occludes the effects of p300

**Fig. 5:**
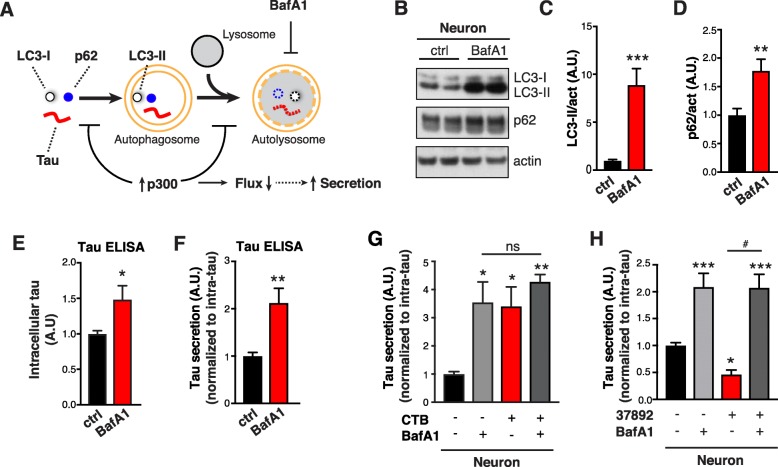
Blocking Autophagic Flux Increases Tau Secretion and Occludes the Effects of p300 **a** Schematic diagram showing autophagic flux and predicted changes in autophagic markers with baflomycin A1 (BafA1) treatment. **b**–**f** BafA1 treatment in primary neurons. **b** Representative immunoblots of LC3-I, LC3-II, p62 and actin in lysates of rat primary neurons treated with BafA1 (10 nM) or control (ctrl, DMSO) for 24 h. Quantification of levels of LC3-II (**c**) and p62 (**d**) relative to actin, normalized to control. **e** Quantification of intracellular tau levels by ELISA, normalized to control. **f** Quantification of tau secretion over 3 h by ELISA, normalized to intracellular tau levels and normalized to control. (**b**–**f**) n = 6 wells from three independent experiments. ***p < 0.001, **p < 0.01, *p < 0.05 by unpaired t test. **g** Quantification of tau secretion over 3 h in primary neurons treated with BafA1 (10 nM), CTB (50 μM) or combined, normalized to intracellular tau levels and normalized to control (CTB- BafA1-). *n* = 6 wells from three independent experiments. **p < 0.01, *p < 0.05, ns, non-significant by one-way ANOVA and Sidak’s multiple comparisons test. **h** Quantification of tau secretion over 3 h in neurons treated with BafA1 (10 nM), 37892 (50 μM) or combined, normalized to intracellular tau levels and normalized to control (37892- BafA1-). n = 6 wells from three independent experiments. *, #*p* < 0.05, ***p < 0.001 by one-way ANOVA and Sidak’s multiple comparisons test. Values are mean ± SEM

The correlation between p300/CBP-induced ALP impairment and excessive tau secretion prompted us to investigate how autophagic flux affects tau secretion. First, we used BafA1 to block lysosomal degradation and inhibit autophagic flux (Fig. 5a). In primary neurons, BafA1 treatment led to accumulation of LC3-II and p62 as expected (Fig. 5 b–d) [42, 43]. Intracellular tau levels were increased by BafA1 treatment (Fig. 4e), indicating that tau is normally degraded by ALP. Remarkably, tau secretion (after normalization to intracellular tau levels) was increased by two-fold in this setting (Fig. 4f Additional file 1: Figure S5 *A*). The majority of the increase was not due to cell death or increased membrane permeability, as shown by unaffected lactate dehydrogenase (LDH) release, minimal caspase-3 cleavage and undetectable α-tubulin release (Additional file 1: Figure S5 *B-E*). Similar effects on tau accumulation and secretion were observed in primary neurons treated with a combination of NH_4_Cl (pH neutralizer) and leupeptin (inhibitor of lysosomal enzymes) (N/L). (Additional file 1: Figure S5 *F*–*J*). Similarly, in human iPSC-derived neurons, treatment with N/L (Additional file 1: Figure S5 *K* and *L*), or vinblastine (Additional file 1: Figure S5 *M* and *N*), which inhibits the fusion of autophagosomes with endosomes and lysosomes [44], promoted secretion of endogenous hTau. Having established that blocking autophagic flux at late steps of ALP increases tau secretion, we asked whether the effect of p300/CBP on tau secretion is mediated by its impairment of autophagic flux. In primary neurons, CTB or BafA1 treatment each increases tau secretion, and co-treatment with CTB and BafA1 did not result in an additional increase in tau secretion compared to the case of single treatment (Fig. 5g). Similarly, while p300 overexpression alone and BafA1 treatment alone both increased tau secretion in HEK293T, there was no synergistic effect of the combination (Additional file 1: Figure S5 *O*). Next, we examined whether the reduction in tau secretion associated with p300 inhibition is mediated through autophagy. Primary neurons treated with p300 inhibitor 37892 alone had reduced tau secretion, as shown previously (Fig. 5h). When autophagic flux was blocked by BafA1 co-treatment in these neurons, the effect of 37892 on tau secretion was masked by the effect of BafA1 treatment (Fig. 5h). Thus, blocking autophagic flux by BafA1 completely abolished the effect of p300 inhibition by 37892 on tau secretion. These results showed that blocking autophagic flux increases tau secretion and occludes the effects of p300, suggesting that ALP impairment is downstream of p300/CBP hyperactivity.

### Enhancing autophagy flux mitigates p300-mediated tau secretion

**Fig. 6:**
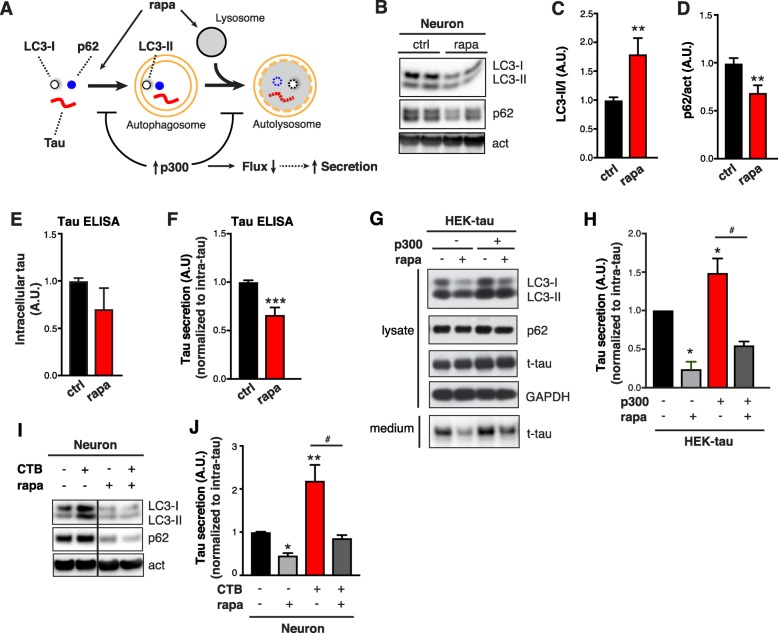
Enhancing Autophagy Flux Mitigates p300-mediated Tau Secretion **a** Schematic diagram showing autophagic flux and predicted changes in autophagic markers with rapamycin (rapa) treatment. **b**–**f** Rapa treatment in primary neurons. **b** Representative immunoblots of LC3-I, LC3-II, p62 and actin in lysates of rat primary neurons treated with rapa (0.25 μM) or DMSO (ctrl) for 24 h. Quantification of levels of LC3-II/I (**c**) and p62 (**d**) relative to actin, normalized to control. **e** Quantification of intracellular tau levels by ELISA, normalized to control. **f** Quantification of tau secretion over 3 h by ELISA, normalized to intracellular tau levels and normalized to control. (**b**–**f**) *n* = 6 wells from three independent experiments. ***p < 0.001, **p < 0.01 by unpaired *t* test. **g**, **h** Rapamycin treatment in HEK293T cells with and without p300 overexpression. **g** Representative immunoblots of LC3-I, LC3-II, p62, total tau (t-tau) and GAPDH in lysate and conditioned medium of HEK293T cells transfected with tau alone or tau+p300. Serum-starved HEK293T cells were treated with DMSO (ctrl) or rapamycin (1 μM) for 24 h. **h** Quantification of t-tau secretion in (**g**) normalized to intracellular levels and normalized to control. *n* = 3 wells from three independent experiments. *, #*p* < 0.05 by one-way ANOVA and Sidak’s multiple comparisons test. **i**, **j** Primary neurons treated with CTB and rapa. **i** Representative immunoblots of LC3-I, LC3-II, p62, and actin in lysates of mouse primary neurons treated with DMSO (ctrl), CTB (50 μM), rapa (0.25 μM) and CTB + rapa. **j** Quantification of tau secretion over 3 h by ELISA of neurons as in (**i**), normalized to intracellular tau levels and normalized to control (CTB- rapa-). *n* = 6 wells from three independent experiments. *, #*p* < 0.05, **p < 0.01 by one-way ANOVA and Sidak’s multiple comparisons test. Values are mean ± SEM

We next examined if promoting autophagic flux affects tau secretion. Rapamycin treatment induces autophagic flux by promoting autophagic induction via mTOR inhibition [45] and enhancing lysosomal degradation [46] (Fig. 6a), resulting in increased LC3-II/I (reflected in reduced LC3-I) and reduced p62 (Fig. 6b–d). In primary neurons, rapamycin treatment led to a trend of reduction in intracellular tau accumulation (Fig. 6e*)*, and significantly reduced tau secretion (Fig. 6f). Next, we tested whether promoting autophagic flux rescues tau secretion induced by p300 overexpression in hTau-expressing HEK293T cells. In the absence of p300 overexpression, rapamycin enhanced autophagic flux, as indicated by an increased turnover of LC3-I to LC3-II (reflected in reduced LC3-I levels) and reduced p62 levels (Fig. 6g, 2^nd^ lane, Additional file 1: Figure S6 *A* and *B*), and reduced tau secretion (Fig. 6 g and h). In cells overexpressing p300, enhancing autophagic flux by rapamycin (Additional file 1: Figure S6 *A* and *B)* abrogated the enhanced secretion of tau (Fig. 6 g and h). Similarly, in primary neurons, enhancing p300/CBP activity by treating with CTB elevated tau secretion, and this was suppressed by co-treatment with rapamycin (Fig. 6 i and j). Together, these results suggest that the effect of p300/CBP on tau secretion is mediated by reduced autophagic flux.

### Inhibition of p300/CBP reduces tau spreading

**Fig. 7:**
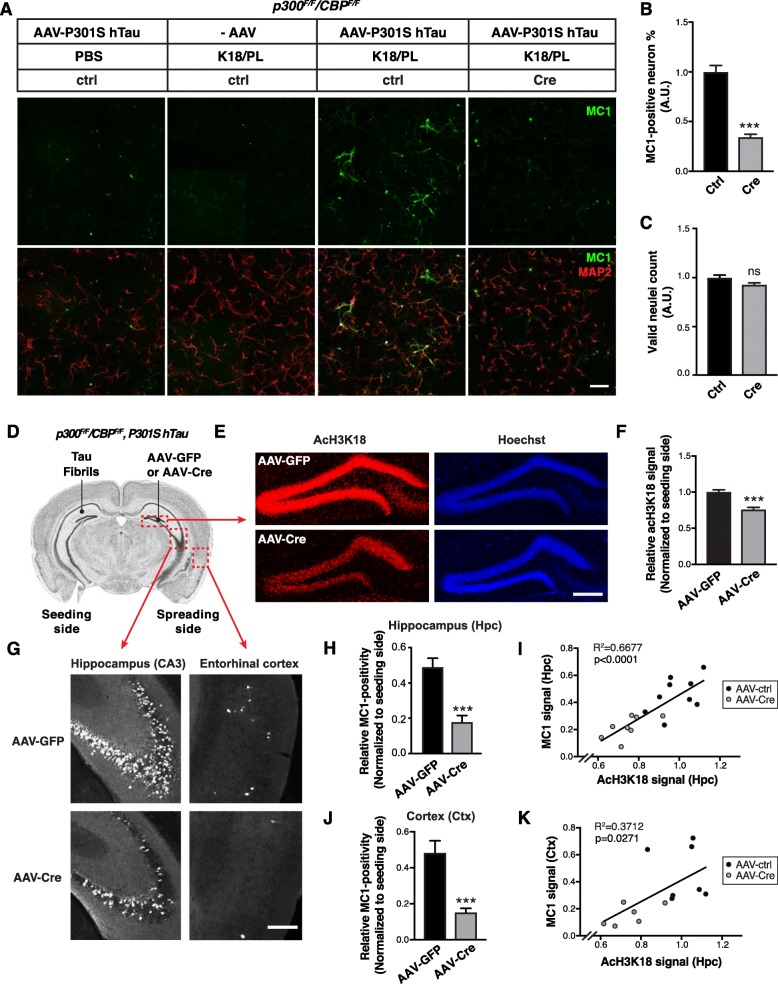
Inhibition of p300/CBP Reduces Tau Spreading **a**–**c** p300/CBP double knockout reduces fibril-induced tau spreading in primary neurons. **a** Representative immunofluorescence staining with MC1 and MAP2 antibody in *p300*^*F/F*^*/CBP*^*F/F*^ primary neurons infected with AAV-P301S hTau and lenti-ctrl or lenti-Cre, and treated with synthetic tau fibrils (K18/PL, 100 nM). Negative controls (PBS-treated, AAV non-infected) are included. Scale bar: 100 μm. **b** Percentage of MC1-positive neurons, normalized to control. ***p < 0.001, unpaired *t* test. **c** Number of valid (live) nuclei, normalized to control. *n* = 9 wells from two independent experiments. **d**–**k** Inhibition of p300/CBP reduces tau spreading in PS19 mice. **d** Schematic diagram of stereotaxic injections in the hippocampus of 3–4 mo PS19 mice carrying *p300*^*F/F*^*/CBP*^*F/F*^. Tau fibrils (K18/PL) were injected into left CA1 (seeding side). AAV-Cre or AAV-GFP were injected into right dentate gyrus (spreading side). **e** Representative images of immunostaining with acH3K18 antibody in the hippocampus after AAV-GFP (control) and AAV-Cre injections. Scale bar: 200 μm. **f** Quantification of acH3K18-positive area (normalized to Hoechst) on the spreading side (AAV-injected) normalized to the seeding (fibril-injected) side of hippocampus. **g** Representative images of immunostaining of MC1 in the hippocampus (CA3) and cortex (entorhinal) after AAV-GFP and AAV-Cre injections. Scale bar: 250 μm. **h, j** Quantification of MC1-positive area of hippocampus (**h**) and cortex (**j**) on the spreading side (AAV-injected) normalized to the seeding (fibril-injected) side. **i, k** Pearson correlation analysis of acH3K18 signal in the AAV-injected hippocampus with MC1 signal in the hippocampus (**i**) and cortex (**k**). *n* = 7 slices from 9 (AAV-GFP) or 8 (AAV-Cre) mice per group. (**f**, **h**, **j**) ***p < 0.001, unpaired *t* test. Values are means ± SEM

Tau secretion is the first step in the process of intercellular tau transmission and the spread of tau pathology. To determine whether p300/CBP affects the spread of pathogenic tau, we used an in vitro model in which synthetic tau fibrils (K18/PL) induce the formation and intercellular spreading of tau aggregates in primary neurons expressing P301S hTau [27]. This seeding-based assay reflects both the initial seeding by tau fibrils and the secondary seeding (propagation) by tau secreted from neurons [27]. MC1 immunostaining is used to detect the pathological, aggregated conformation of tau [47] (Fig. 7a). Double knockout of p300/CBP markedly reduced the number of neurons bearing MC1-positive tau aggregates without affecting neuron number (Fig. 7a–c). Reduced MC1 signal was also observed in p300/CBP double heterozygous neurons, in the absence of changes in neuron number and neurite length (Additional file 1: Figure S7 *A*–*E*). A similar effect was observed in primary neurons treated with 37892 (Additional file 1: Figure S7 *F*–*I*). Thus, inhibiting p300/CBP reduces seed-induced tau pathology in vitro.

To determine whether p300/CBP inhibition reduces tau spreading in vivo, we simultaneously injected tau fibrils into the left hippocampus (CA1) and AAV-Cre or AAV-GFP into the right hippocampus (dentate gyrus) of 4–5-month-old (mo) PS19 mice carrying *p300*^*F/F*^ and *CBP*^*F/F*^ (Fig. 7d). Deletion of p300/CBP in the right hippocampus resulted in reduced staining for acH3K18 (Fig. 7 e and f). Consistent with previous studies [4], injection of tau fibrils markedly increased MC1-positive tau pathology in the ipsilateral and contralateral hippocampi within 4 weeks in PS19 mice expressing AAV-GFP (Fig. 7g). Non-transgenic littermates injected with tau fibrils did not have detectable MC1 signal in 4 weeks (Additional file 1: Figure S8 *A*). In PS19 mice, Cre-mediated p300/CBP deletion significantly reduced the spread of MC1-positive tau to the contralateral hippocampus and cortex (Fig. 7g, h, j, Additional file 1: Figure S8 *B* and *C*). Remarkably, the extent of tau spreading quantified by MC1 positivity correlated with p300/CBP activity in AAV-injected hippocampus, measured by acH3K18 signal (Fig. 7 i and k). Thus, inhibition of p300/CBP has the potential to reduce the spread of tau pathology in vivo.

## Discussion

Our study shows that p300/CBP acetyltransferase activity is aberrantly increased in tauopathy in humans and mice, and correlates with ALP impairment. In primary neurons and human iPSC-derived neurons, inhibition of p300/CBP enhanced autophagic flux and suppressed tau secretion, and activating p300/CBP had the inverse effect. Moreover, p300/CBP inhibition strongly reduced the pathogenic spread of tau inclusions in vivo. Through detailed mechanistic dissections, we showed that p300/CBP promotes tau secretion by inhibiting autophagic flux. These findings establish a novel connection between p300/CBP-mediated ALP impairment and tau propagation.

p300/CBP, best known as transcriptional cofactor, was found to be a master regulator of AD progression, revealed in an unbiased system approach analysis of gene expression profiles from laser-captured neurons from AD and controls subjects [16]. Indeed, we found the levels of acetylated H3K18, the canonical substrate of p300, are increased in the CSF of patients with AD, consistent with elevated p300 activity as previously reported [16, 48]. Interestingly, other acetylation sites that are independent of p300/CBP, such as H4K16 [28], were found to be reduced in AD brain [49], highlighting the specific elevation of histone acetylation that depends on p300/CBP activity. How p300/CBP becomes hyperactive in diseased brains is unclear. We found that p300/CBP activity is increased in 3 mo PS19 mice compared with their wild-type littermates, suggesting that hyperactivity of p300/CBP is an early event in tauopathy, before the accumulation of insoluble tau. p300/CBP hyperactivity in turn enhances tau accumulation, secretion and spreading, resulting in a feedforward vicious cycle.

Tau homeostasis requires degradation by the UPS and ALP (reviewed in [6]). Here, we found that elevated p300/CBP activities correlated with ALP impairment in mice with tauopathy. Autophagy-mediated tau degradation is known to be inhibited by pathogenic tau point mutations (P301L, A152T) and post-translational modifications [50]. Furthermore, autophagic and endolysosomal dysfunction is accompanied by accumulation of immature AVs in the brains of AD patients [16, 30, 32, 51]. Our findings implicate p300/CBP hyperactivity as a key player in the impairment of autophagic flux that slows the turnover of AVs through lysosomal degradation. Previous studies showed that p300 acetylates the autophagy components Atg5, Atg7, Atg8, and Atg12, and that knockdown of p300 reduces p62 levels and increases LC3-II levels in Hela cells [33]. In neurons, we showed that p300/CBP knockout reduced p62 levels and increased ratio of LC3-II/I ratio, similar to Hela cells and fully consistent with increased autophagy flux. However, different from that in Hela cells, p300/CBP knockout in neurons resulted in reduced total LC3 (including LC3-I and LC3-II) levels. This discrepancy is likely due to differences in basal levels of autophagy in cancer cell lines versus postmitotic cells, such as neurons. Indeed, neurons have been shown to exhibit more efficient clearance of newly forming autophagosomes by lysosomes [31]. In addition, p300/CBP activation results in accumulation of LC3 and p62, suggesting a blockage in late stage ALP. Together, our results suggest that p300/CBP reduces autophagic flux at both the induction stage and maturation/degradation stage (Fig. 6a).

We showed that p300/CBP activation promotes tau secretion, likely via inhibition of ALP. Blocking lysosomal degradation or the fusion of autophagosomes with lysosomes markedly increased tau secretion, whereas promoting autophagic flux with rapamycin reduced tau secretion. Thus, tau secretion could be an alternative clearance mechanism to maintain overall cellular proteostasis when degradative pathways are impaired or overloaded. We found that increasing autophagic flux mitigates tau secretion in cells with enhanced p300/CBP activity, and blocking autophagic flux abolished the beneficial effect of p300/CBP inhibition. These findings provide strong evidence that the effects of p300/CBP on tau secretion are mediated via its modulation of ALP.

How does p300/CBP hyperactivation inhibit ALP and promotes tau secretion? It was recently reported that p300-mediated acetylation of beclin 1, VPS34, and SIK2 inhibits autophagy initiation, autophagosome maturation, and endocytic trafficking [34, 52, 53], suggesting that these proteins may play a role in regulating secretion of tau and potentially other proteins. Secondly, as p300/CBP regulates histone acetylation, the mechanism could also involve epigenetic changes in the autophagy-relevant transcriptome and synaptic plasticity [21, 54, 55]. Finally, acetylation of tau itself by p300/CBP increases tau toxicity, which could lead to cell damage such as impaired cytoskeleton stability and membrane integrity, and in turn contribute to elevated secretion.

The cellular trafficking through which tau is secreted is unknown. Secretion of other proteins have been attributed to autophagy-mediated unconventional secretion pathway without the involvement of Golgi-ER complex, such as IL1β [14, 15]. In Parkinson’s disease, α-synuclein is secreted through a similar mechanism, in which accumulated autophagic intermediates such as autophagosome, autolysosome and multivesicular bodies (MVBs) fuse with the plasma membrane and release α-synuclein [56]. We found that increased tau secretion is associated with accumulation of LC3-II and p62, markers of intermediate AVs. However, it was previously reported that LC3-labeled autophagosomes do not appear to be enriched in tau [38], and indeed we found tau inclusions largely reside in the cytosol without specific colocalization with a certain type of autophagic vesicles. In addition, Atg5 knockout neurons, which lack LC3-II-positive autophagosomes, secrete even more tau (data not shown), suggesting that autophagosomes are not the (only) vesicles responsible for tau secretion. In Atg5 knockout neurons, autophagosomes do not form, imposing a heavier tau burden for other vesicles in ALP such as MVB, late endosomes and lysosomes, which could lead to increased tau secretion. Indeed, tau has been found in exosomes isolated from cultured neurons and CSF, implicating an MVB-mediated release mechanism in tau secretion [10, 57]. It was also reported that Rab7A, a small GTPase involved in endosomal trafficking, regulates tau secretion, indicating that a late endosomal compartment is involved [58]. Finally, as the last step in ALP, lysosomes can undergo exocytosis, a process resembling synaptic vesicle release regulated by TFEB [59], which could also mediate tau secretion when autophagy is impaired. In support of the hypothesis that later-stage intermediate vesicles in ALP mediate tau secretion, we and others [16] found accumulation of GVB-like structures with molecular signature of late endosome and lysosome in P301S hTau transgenic mouse brain (data not shown).

Pathogenic tau species could differ in their effects on different autophagy pathways (macroautophagy, chaperone-mediated macroautophagy and endosomal microautophagy) [50], and there is complex crosstalk between autophagy pathways and endolysosomal pathways [60]. For example, autophagy induction promotes the fusion of MVBs with lysosomes and inhibits exosome release [61]. It was recently found that rapamycin induces autophagic flux via TRPML1-TFEB pathway on lysosomes, independent of mTORC-ULK1 inhibition [46]. In line with this, we found that rapamycin induces autophagic flux and reduces tau secretion, both in WT cells and in cells with p300 activation. Thus, fine-tuning of ALP at multiple steps can push the system to reach a new balance between degradation and secretion of the cargo.

Increased secretion of tau into the extracellular space could underlie tau propagation to different brain areas, the spread of pathology, and disease progression. Here we showed that p300/CBP inhibition reduced both endogenous tau accumulation and the spread of aggregated tau pathology. Promoting autophagy by rapamycin reduces tau pathology in mouse models [62, 63] and reduces tau spreading in a seeding based cellular assay of tauopathy [64]. Our results suggest that inhibition of p300/CBP promotes autophagic flux, enhances tau degradation and reduces tau secretion, thereby attenuating tau spreading. p300/CBP inhibition also reduces tau acetylation, which in turn enhances tau clearance and reduces tau accumulation. Thus, the attenuated spread of tau pathology we observed with inhibition of p300/CBP likely resulted from reduction of both intracellular tau accumulation and extracellular tau secretion. Tau propagation is thought to be caused by soluble tau species, either monomeric or oligomeric [65, 66]. However, it remains to be determined whether p300/CBP affects posttranslational modification of transmitted tau species, or tau seed uptake. In addition, p300/CBP’s other effects on neurons [21], including neuronal plasticity [55, 67] could also indirectly contribute to modulation of tau spreading. Finally, glial cells may contribute to tau spreading [68], and the p300/CBP-autophagy pathway could also be involved in these cells.

## Conclusions

In summary, our findings that p300/CBP hyperactivity impairs ALP, promotes tau secretion and pathogenic propagation, supports targeting p300/CBP-ALP as a novel strategy to enhance autophagy and to counteract tau pathogenesis. To this end, we performed an HTS for new p300 inhibitors in the context of tau, and identified a new compound that reduces tau acetylation and accumulation, and in the meantime, enhances ALP and reduces tau secretion and propagation. The in vivo efficacy and further development of this compound warrants future investigation.

## Supplementary information

**Additional file 1: Figure S1.** p300/CBP activity measured by AcH3K18 shows an increase in 3 mo PS19 mice. **Figure S2.** p300 overexpression in HEK293T cells reduces autophagic flux. **Figure S3.** CTB treatment increase tau secretion without affecting cytotoxicity or Aβ release. **Figure S4.** Design of the high-throughput screen and additional characterization of 37892. **Figure S5.** Blocking ALP by BafA1, N/L and vbl increases tau secretion in neurons. **Figure S6.** Rapamycin promotes autophagic flux in HEK293T cells. **Figure S7.** Inhibition of p300/CBP by heterozygous floxed deletion and 37892 reduces seed-induced tau pathology in vitro. **Figure S8.** Full area view of hippocampus and cortex section showing MC1-positive tau pathology in fibril- and AAV-injected PS19 mice carrying p300^F/F^ /CBPF/F

## Data Availability

The datasets used and/or analyzed during the current study are available from the corresponding author on reasonable request.

## Abbreviations

A.U.: Artificial unit
AD: Alzheimer’s disease
ALP: The autophagy-lysosomal pathway
AVs: Autophagic vacuoles
BafA1: Bafilomycin A1
CSF: Cerebrospinal fluid
MVBs: Multivesicular bodies
UPS: The ubiquitin-proteasomal system
WT: Wild-type
