# Supplementary material for: Promoting tau secretion and propagation by hyperactive p300/CBP via autophagy-lysosomal pathway in tauopathy
Source: Mol Neurodegener. 2020 Jan 6;15:2. doi: 10.1186/s13024-019-0354-0 (PMC6945522; doi:10.1186/s13024-019-0354-0)
Supplement: Supplementary file 1 — Additional file 1: Figure S1. p300/CBP activity measured by AcH3K18 shows an increase in 3 mo PS19 mice. Figure S2. p300 overexpression in HEK293T cells reduces autophagic flux. Figure S3. CTB treatment increase tau secretion without affecting cytotoxicity or Aβ release. Figure S4. Design of the high-throughput screen and additional characterization of 37892. Figure S5. Blocking ALP by BafA1, N/L and vbl increases tau secretion in neurons. Figure S6. Rapamycin promotes autophagic flux in HEK293T cells. Figure S7. Inhibition of p300/CBP by heterozygous floxed deletion and 37892 reduces seed-induced tau pathology in vitro. Figure S8. Full area view of hippocampus and cortex section showing MC1-positive tau pathology in fibril- and AAV-injected PS19 mice carrying p300F/F /CBPF/F [file 13024_2019_354_MOESM1_ESM.pdf]

Figure S1. (Related to Figure 1)

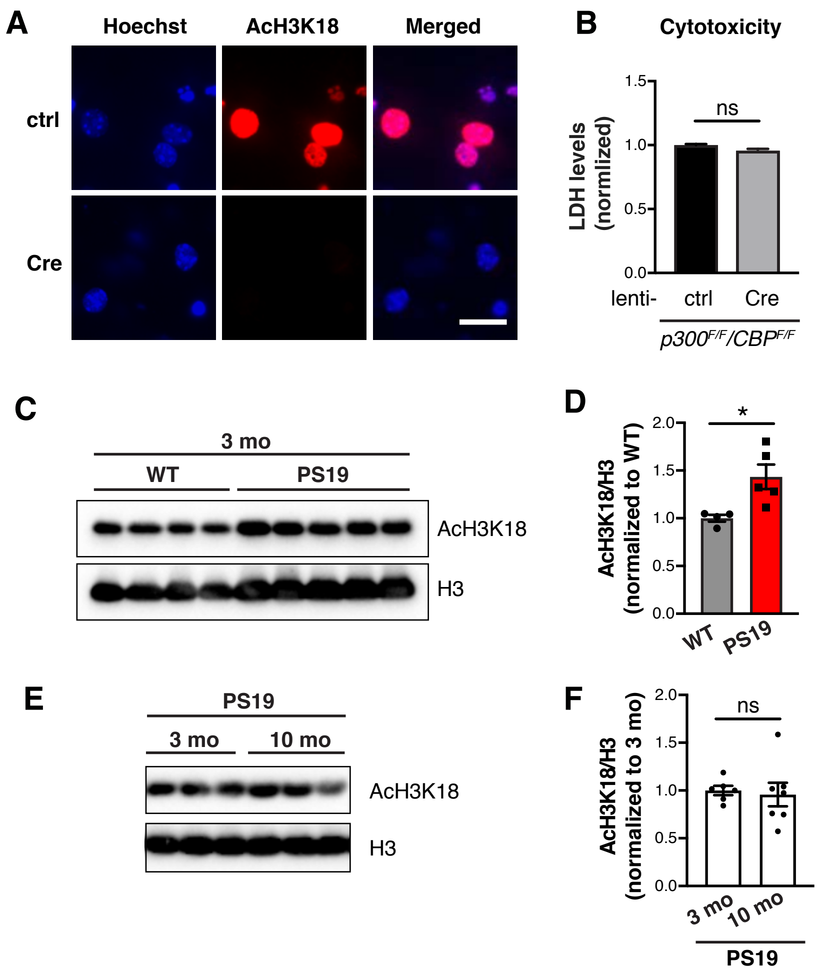

(A) Representative immunofluorescence staining with anti-acH3K18 antibody and Hoechst in *p300*$^{F/F}$/*CBP*$^{F/F}$ primary neurons infected with lenti-ctrl or lenti-Cre. Scale bar: 20 µm.
(B) Cytotoxicity in an LDH release assay, normalized to ctrl. n=4 wells from two independent experiments.
(C-F) p300/CBP activity measured by AcH3K18 levels in hippocampi of young (3 mo) PS19 mice vs. wild-type (WT) littermates (C, D), and young (3 mo) vs. old (10 mo) PS19 mice (E, F). *p<0.05, ns, non significant, unpaired *t*-test. Values are mean ± SEM.

Figure S2 (Related to Figure 2)

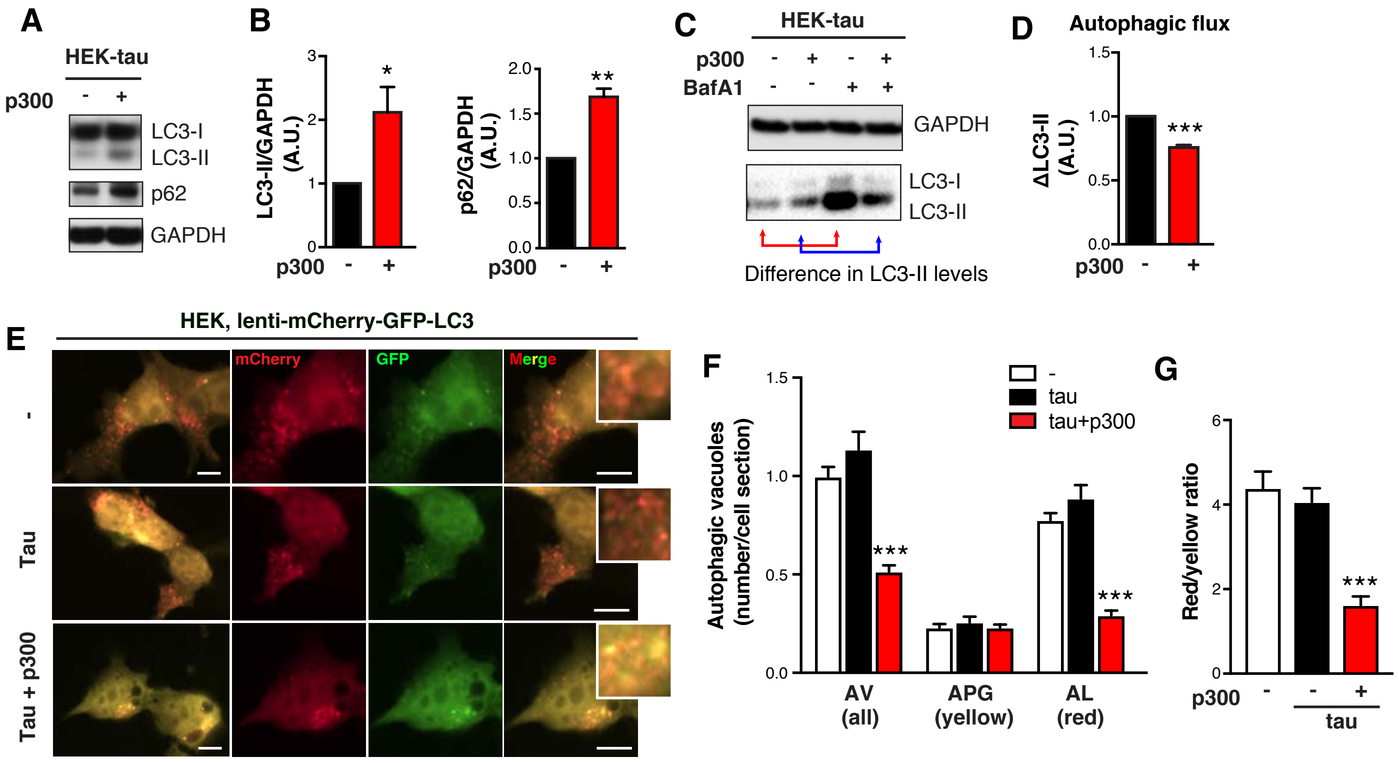

(A, B) p300 overexpression in HEK293T cells increases LC3-II and p62 accumulation. (A) Representative immunoblot of LC3-I, -II, SQSMT/p62 and GAPDH in lysates of HEK293T cells transfected with tau alone or tau+p300. HEK293T cells were serum-starved for 48 h after transfection. (B) Quantification of levels of LC3-II and p62 relative to GAPDH, normalized to control (tau alone). n=3 wells from three independent experiments. *p<0.05, **p<0.01 by unpaired t test.
(C, D) p300 overexpression in HEK293T cells reduces autophagic flux. (C) Representative immunoblots of LC3-I, -II, and GAPDH in lysates of HEK293T cells transfected with tau alone or tau+p300 and treated with BafA1 (10 nM, 24 h) or DMSO in serum-free medium. (D) Quantification of autophagic flux by the difference (increase) of LC3-II in response to BafA1, normalized to control (p300-, BafA1-). n=4 wells from two independent experiments. *p<0.05, ***p<0.001 by unpaired t test.
(E–G) HEK293T cells stably expressing mCherry-GFP-LC3 are transfected with vector (-), tau, or tau+p300, and serum starved for 4 h to induce autophagy. (E) Representative images of mCherry and GFP fluorescent signal. Scale bar: 10 µm. (F) Quantification of autophagic vesicles (AV). Autophagosomes (APG) are identified as yellow vesicles retaining both mCherry and GFP fluorescence. Autolysosomes (AL) are identified as red vesicles in which GFP fluorescence is quenched by the low pH in lysosomes. ***p<0.001, two-way ANOVA, Tukey-Kramer post hoc analysis. (G) Ratio of the number of red vesicles to yellow vesicles per cell. ***p<0.001, one-way ANOVA, Tukey-Kramer post hoc analysis. (F, G) From two independent experiments, n=51 cells (-), n=24 cells (tau), and n=53 cells (tau+p300). Values are mean ± SEM.

Figure S3. (Related to Figure 3)

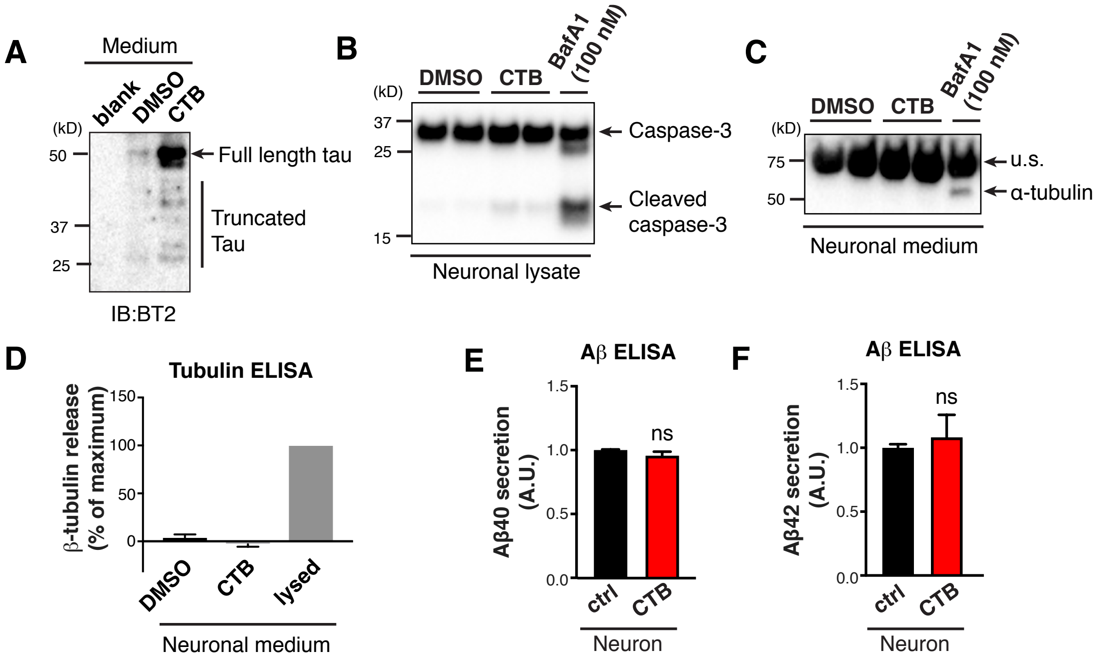

(A) Immunoblot with BT2 antibody in blank or conditioned media of primary neurons treated with DMSO or CTB (50 uM). Both full length mouse tau and trancated tau were detected. (B) Representative immunoblot of caspase-3 (uncleaved and cleaved) in DMSO and CTB-treated primary mouse neuron lysates. 100 nM BafA1 treatment leading to cell toxicity is included as a positive control for caspase-3 cleavage. (C) Representative immunoblot of α-tubulin in conditioned media of primary mouse neuron treated with DMSO and CTB. 100 nM BafA1 treatment leading to cell toxicity is included as a positive control for α-tubulin release. Media was concentrated by 10 folds. u.s. indicates a unspecific band. (D) Quantification of mouse β-tubulin in the conditioned medium of primary neurons treated with DMSO and CTB by ELISA. n=2 wells from 2 independent experiments. Triton-X lysed neuronal medium is included as a positive control for maximum β-tubulin release. (E, F) Quantification of levels of endogenous Aβ40 (E) and Aβ42 (F) in the conditioned medium of neurons treated with CTB or DMSO (ctrl) by ELISA, normalized to control. n=6 wells from three independent experiments. ns, non-significant by unpaired t test. Values are mean ± SEM.

Figure S4. (Related to Figure 4)

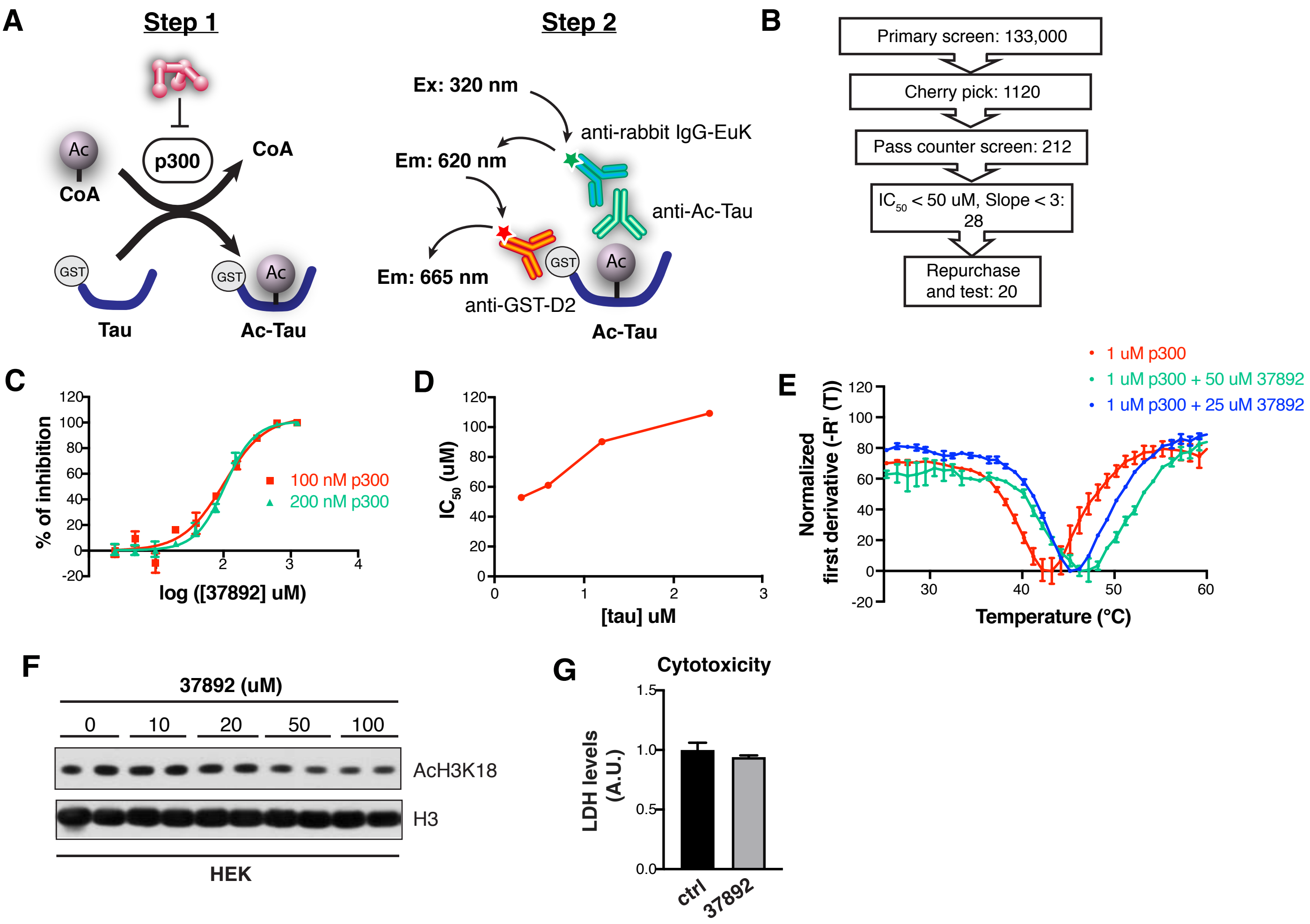

(A) Diagram of the homogeneous time-resolved fluorescence assay. Step 1, the enzymatic reaction: p300 transfers an acetyl group from acetyl-CoA to tau, producing CoA and ac-tau. Step 2, detection: a rabbit ac-tau–specific antibody (mAB359) recognizes ac-tau, the europium cryptate-labeled anti-rabbit IgG-EuK (donor) binds to mAB359, and the anti-GST-D2 (acceptor) captures GST-tau. When tau is acetylated, the Eu donor and D2 acceptor are brought into proximity, allowing Forster resonance energy transfer (FRET) to occur. FRET is detected as a long-lived fluorescence signal at 665 nm. The fluorescence signal ratio 665/620 nm is proportional to the extent of ac-tau in the solution. (B) Work flow of the high-throughput screen. (C) Orthogonal MMBC assay confirms the inhibitory activity of 37892, albeit at a lower IC50 of 100 µM. IC50 of 37892 remained the same under different p300 concentrations, indicating that aggregation is not a likely mechanism of inhibition. (D) Dose-response curves of 37892 under different tau concentrations. (E) Differential scanning fluorimetry shows that 37892 binds to p300. The Tm of p300 increases from 42.5°C to 45.4°C and 47.2°C when 25 µM and 50 µM 37892 was added, respectively. (F) Representative immunoblots of acH3K18 and H3 in histone extracts of HEK293T cells treated with increasing doses of 37892 for 24 h. (G) Cytotoxicity in an LDH release assay, normalized to ctrl. n=4 wells from two independent experiments.

Figure S5. (Related to Figure 5)

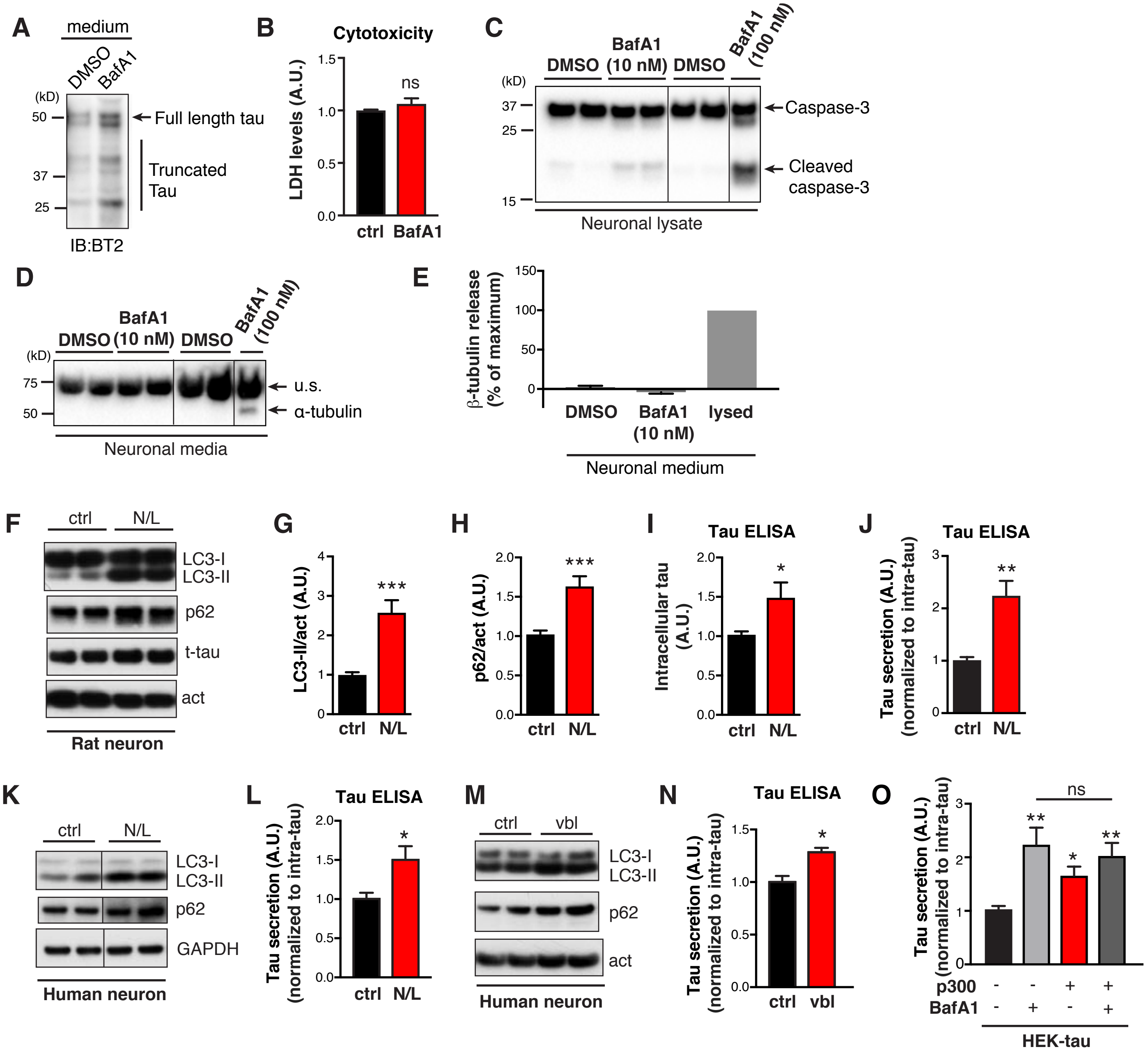

(A) Immunoblot with BT2 antibody in conditioned media of primary neurons treated with DMSO or BafA1 (10 nM). Both full length mouse tau and trancated tau were detected. (B) Quantification of cytotoxicity in hTau-expressing primary neurons treated with BafA1 (10 nM) using LDH release assay, normalized to ctrl. n=6 wells from 3 independent experiments. ns, non-significant by unpaired t test. (C) Representative immunoblot of caspase-3 (uncleaved and cleaved) in DMSO and BafA1 (10 nM)-treated primary mouse neuron lysates. 100 nM BafA1 treatment leading to cell toxicity is included as a positive control for caspase-3 cleavage. (D) Representative immunoblot of α-tubulin in conditioned media of primary mouse neuron treated with DMSO and BafA1 (10 nM). 100 nM BafA1 treatment leading to cell toxicity is included as a positive control for α-tubulin release. Media was concentrated by 10 folds. u.s. indicates an unspecific band. (E) Mouse β-tubulin ELISA in conditioned medium of primary neurons treated with DMSO and BafA1 (10 nM). n=3 wells from 2 independent experiments. Triton-X lysed neuronal medium is included as a positive control for maximum β-tubulin release. (F–J) Blocking autophagic flux with NH_4Cl/leupeptin (N/L) increases tau secretion in primary neurons expressing hTau. (F) Representative immunoblots of LC3-I, LC3-II, p62, and actin in lysates of rat primary neurons infected with AAV-P301S hTau, after treatment with N/L (20 mM NH_4Cl and 200 μM leupeptin) for 24 h. Quantification of levels of LC3-II (G) and p62 (H) relative to actin, normalized to control. (H) Quantification of intracellular tau levels by ELISA and normalized to control. (I) Quantification of tau secretion over 3 h, normalized to control. n=6 wells from 3 independent experiments. *p<0.05, **p<0.01, ***p<0.001, ns, non-significant by unpaired t test. (K–O) N/L and vinblastine (vbl) block autophagic flux and increases tau secretion in human-iPSC-derived neurons. (K, M) Representative immunoblot of LC3-I, LC3-II, p62, and GAPDH or actin in lysates of 8–10-week-old human neurons, after 24 h of treatment with DMSO (ctrl), N/L (20 mM NH_4Cl and 200 μM leupeptin), or vinblastine (vbl, 5 μM). (L, N) Quantification of tau secretion with N/L or vbl treatment, normalized to control. n=4 wells from two independent experiments. *p<0.05, unpaired t test. (O) Quantification of t-tau secretion in HEK293T cells with and without p300 overexpression treated with BafA1 (10 nM), normalized to control (p300- BafA1-). n=7 wells from three independent experiments. *p<0.05, **p<0.01, ns, non-significant by one-way ANOVA and Sidak's multiple comparisons test. Values are mean ± SEM.

Figure S6. (Related to Figure 6)

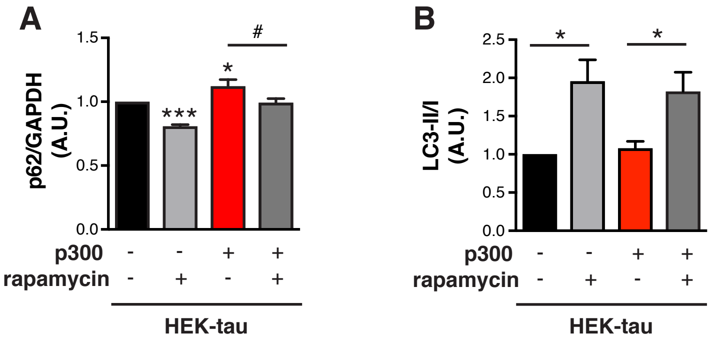

Quantification of p62 levels (A) and LC3-II/I ratio (B) in lysates of HEK293T cells transfected with tau alone or tau + p300 and treated with DMSO (ctrl) or rapamycin (1 µM) for 24 h. n=3 wells from three independent experiments. *, #p<0.05, ***p<0.001 by one-way ANOVA and Sidak's multiple comparisons test. Values are mean ± SEM.

Figure S7. (Related to Figure 7)

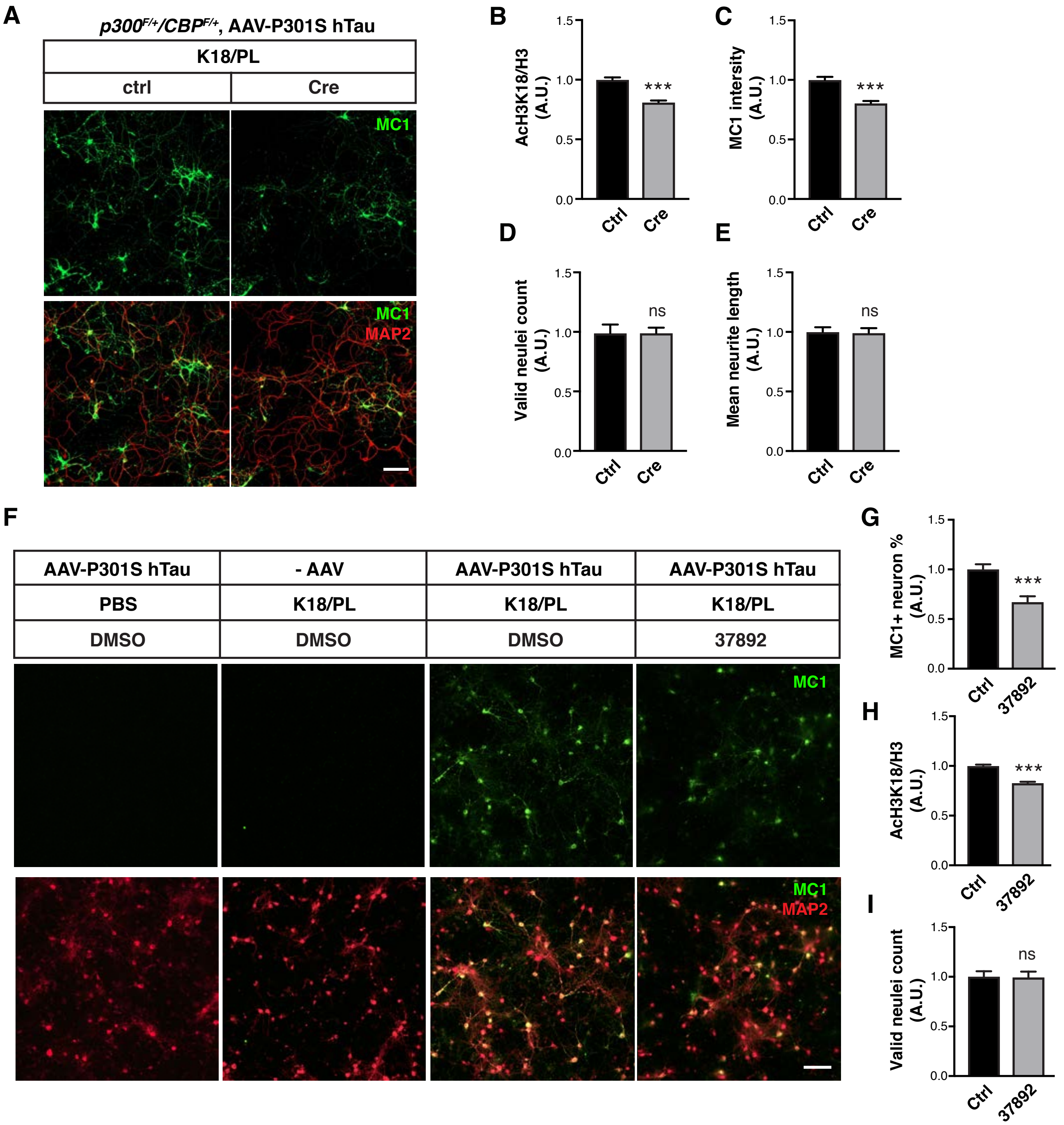

(A–E) p300/CBP heterozygous knockout reduces fibril-induced tau spreading in primary neurons. (A) Representative immunofluorescence staining with MC1 and MAP2 antibody in $p300^{F/+}/CBP^{F/+}$ primary neurons infected with AAV-P301S hTau and lenti-control or lenti-Cre, and treated with synthetic tau fibrils (K18/PL, 100 nM). Scale bar: 100 µm. (B–E) Quantification of acH3K18 signal (B), MC1 intensity (C), number of valid (live) nuclei (D), and mean neurite length (E), normalized to control. ***p<0.001, ns, non-significant by unpaired t test. n=12 wells from two independent experiments. Values are mean ± SEM.
(F–I) 37892 treatment reduces fibril-induced tau spreading in primary neurons. (F) Representative immunofluorescence staining with MC1 and MAP2 antibody in primary rat neurons infected with AAV-P301S hTau and treated with synthetic tau fibril (K18/PL, 100 nM) and 37892 (50 µM) or DMSO (control). Negative controls (PBS-treated, AAV non-infected) are included. Scale bar: 100 µm. (G) Quantification of percentage of MC1-positive neurons, normalized to control. (H) Quantification of acH3K18 signal intensity relative to Hoechst and normalized to control. n>800 cells/treatment. (I) Number of valid (live) nuclei, normalized to control. n=13 fields from two independent experiments. ***p<0.001, ns, non-significant by unpaired t test. Values are mean ± SEM.

Figure S8. (Related to Figure 7)

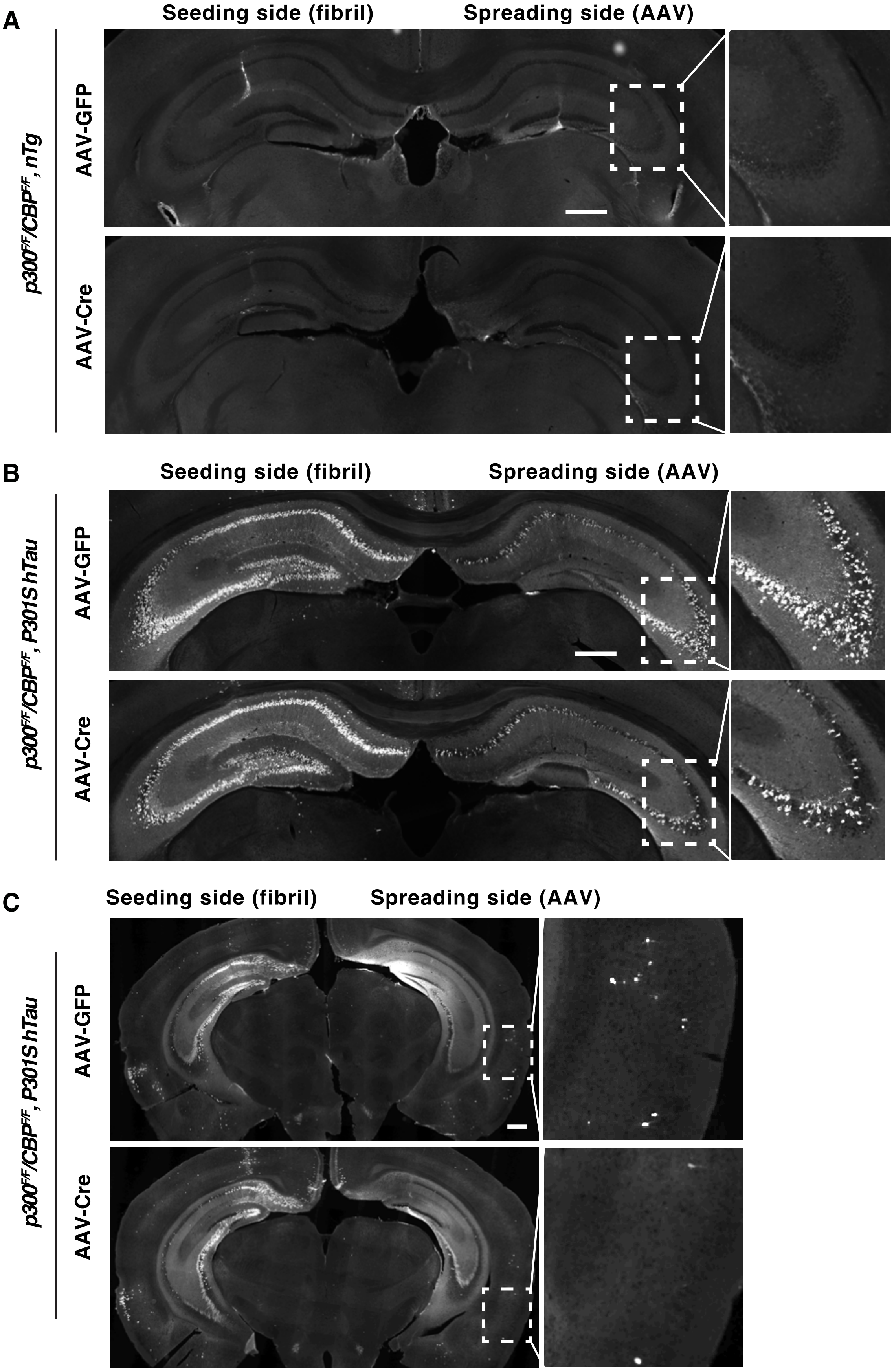

(A) Representative images of immunostaining with MC1 antibody in the hippocampus of non-transgenic (nTg) $p300^{F/F}/CBP^{F/F}$ mice after AAV-GFP and AAV-Cre injections. Scale bar: 500 µm.
(B) Representative images of immunostaining with MC1 antibody in the hippocampus and enlarged CA3 area of PS19 mice carrying *p300*$^{F/F}$*/CBP*$^{F/F}$ after AAV-GFP and AAV-Cre injections. Scale bar: 500 µm.
(C) Representative images of immunostaining with MC1 antibody in the whole brain and enlarged entorhinal cortex area of PS19 mice carrying *p300*$^{F/F}$*/CBP*$^{F/F}$ after AAV-GFP and AAV-Cre injections. Scale bar: 500 µm.

Supplementary Table 1: Patient sample information

PIDN	Diagnosis	Ab421	ptau1	ptau1/Ab421
8964	AD	398	30	0.07537688
13838	AD	234.63	44.07	0.18782764
21339	AD	NA	NA	NA
15032	AD	207.39	37.08	0.17879358
16407	AD	NA	NA	NA
18015	AD	175.38	60.32	0.34393888
8886	AD	165	108	0.65454545
20734	AD	NA	NA	NA
10619	AD	134.17	49.53	0.36915853
20029	AD	NA	NA	NA
20729	AD	NA	NA	NA
19415	AD	116.9	23.11	0.19769033
20596	AD	NA	NA	NA
5064	CN	NA	NA	NA
767	CN	NA	NA	NA
21513	CN	NA	NA	NA
14704	CN	NA	NA	NA
9822	CN	551.09	15.94	0.0289245
6857	CN	379.44	28.46	0.07500527
21297	CN	265.8	22.8	0.08577878
7938	CN	NA	NA	NA
23613	CN	367.09	17.6	0.04794465
22517	CN	282.07	19.09	0.06767824
16938	CN	79.94	19.45	0.24330748
12436	CN	188	8	0.04255319
21514	CN	155.14	17.29	0.11144772
21274	CN	169	36	0.21301775
